# Concentrated Rhamnolipid Formulations: Bridging Chemodiversity to Structure, Flow Behavior, and Functionality

**DOI:** 10.1021/acssuschemeng.5c09437

**Published:** 2025-12-17

**Authors:** Matilde Tancredi, Carlo Carandente Coscia, Michela Buonocore, Alessandro Cangiano, Maria Michela Salvatore, Lorenzo Veronico, Delia Picone, Anna Maria D’Ursi, Manuela Grimaldi, Maria Francesca Ottaviani, Stefano Guido, Luigi Paduano, Luigi Gentile, Gerardino D’Errico

**Affiliations:** a Department of Chemical Sciences, 9307 University of Naples Federico II, Complesso Universitario di Monte Santangelo, Via Cintia 4, Naples I-80126, Italy; b Consorzio Interuniversitario per lo Sviluppo dei Sistemi a Grande Interfase (CSGI), Via della Lastruccia 3, Florence I-50019, Italy; c Department of Veterinary Medicine and Animal Production, 9307 University of Naples Federico II, Via Federico Delpino 1, Naples I-80137, Italy; d Department of Chemistry, University of Bari “Aldo Moro”, Via Orabona 4, Bari I-70126, Italy; e Department of Pharmaceutical Sciences, University of Salerno, via Ponte Don Melillo, Fisciano I-84084, Italy; f Department of Pure and Applied Sciences, University of Urbino “Carlo Bo”, Via Saffi 2, Urbino I-61029, Italy; g Department of Chemical, Materials and Production Engineering, 9307 University of Naples Federico II, P.le Tecchio 80, Naples I-80125, Italy

**Keywords:** eco-sustainable detergents, ultraconcentrated formulations, biosurfactants, congener mixture, self-assembly, rheology

## Abstract

The development of ultraconcentrated biobased formulations is one of the latest frontiers in sustainable product design. This study demonstrates that biosurfactants are well suited to the scope with their natural chemodiversity being a key factor in optimizing formulation structure and function. The composition of a low-cost commercial rhamnolipid sample was investigated by using nuclear magnetic resonance and mass spectrometry. The results revealed a complex mixture of congeners with a predominance of dirhamnolipids and double-tailed species and the presence of long-chain free fatty acids. Polarized optical microscopy, small-angle X-ray scattering, and electron paramagnetic resonance show that this rhamnolipid sample, in aqueous solution, forms ellipsoidal micelles with a highly hydrophobic core, whose dimensions are almost insensitive to concentration (up to 65 wt %) and temperature (up to 50 °C). Analysis of the results hints that a fundamental role in tuning the system behavior is played by the specific rhamnolipid congener composition and by the free fatty acids acting as cosurfactants. At concentrations exceeding 65 wt %, small domains with different supramolecular ordered structures form, suggesting congener segregation. This aggregation behavior explains the preserved low viscosity and good cleaning efficiency. Thus, rhamnolipids are established as valuable candidates for the design of innovative, sustainable formulations.

## Graphical abstract

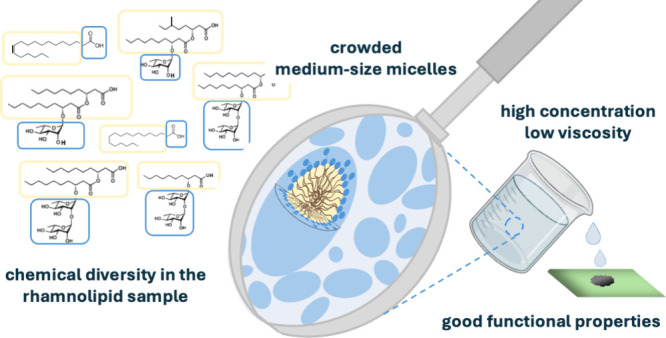

## 1 Introduction

The evolution of the surfactant-based formulation industry since the beginning of the present century has been characterized by two predominant trends, both of which are driven by the objective of enhancing eco-sustainability while maintaining functional performance and economic profit. First, ultraconcentrated (water-poor) liquid formulations have achieved a significant market presence, motivated by the benefits of reducing plastic packaging and enhancing transportation efficiency.^1^ Second, there has been a notable increase in the use of biobased surfactants, which have proven to be more biodegradable and eco-sustainable than conventional synthetic ones.^2^ These developments have been made possible by intensive scientific and technological research that has achieved significant results.

Concerning ultraconcentrated liquid surfactant mixtures, research endeavors have centered on the engineering of surfactants, or surfactant mixtures, capable of forming liquid solution with low viscosity at elevated concentrations.^3^ A possible strategy was found to be the introduction of a single short side chain in the surfactant tail.^4^ On the other hand, research on biobased surfactants has focused on surfactants synthesized from renewable materials. Recent studies have shifted toward natural amphiphiles obtained through microbial fermentation, known as biosurfactants, which minimize the necessity for additional chemical processing.^5^

This paper aims to establish the scientific basis for the convergence of the aforementioned trends, determining whether the conditions exist for the use of biosurfactants in ultraconcentrated formulations. In pursuit of this ambitious objective, the morphological, microstructural, and rheological properties of concentrated aqueous mixtures (i.e., up to the solubility limit) of biosurfactants are investigated. In fact, most previous studies on the self-aggregation of biosurfactants in aqueous mixtures have been carried out under dilute to semidilute conditions, sporadically exceeding 10 wt %,^6^ and only recent studies have addressed the phase behavior in concentrated mixtures.^7,8^ This study centers on rhamnolipids, the biosurfactants closest to practical implementation.^9^ Their technological production from eco-sustainable raw materials, such as agri-food waste, has been well optimized, and large-scale production has also commenced. Rhamnolipids are multifunctional molecules that, in addition to their excellent ability to efficiently reduce surface and interfacial tension, are widely recognized for their antimicrobial, anti-inflammatory, and immunomodulatory properties, which represent significant added value. For this reason, rhamnolipids have been proposed in many applications from pharmaceuticals, food design, cosmetics and personal care, agricultural, oil recovery, and water treatment.^9^

Rhamnolipids are amphiphilic glycolipids biosynthesized by a wide range of microbial species, including bacteria, yeasts, and fungi, but predominantly associated with *Pseudomonas aeruginosa* and other members of the *Pseudomonas* genus.^9^ These compounds feature one or two l-rhamnose units, which are linked by an α-1,2-glycosidic bond, and one or two β-hydroxy fatty acid chains in the R configuration; see Figure 1.

**Figure 1:**
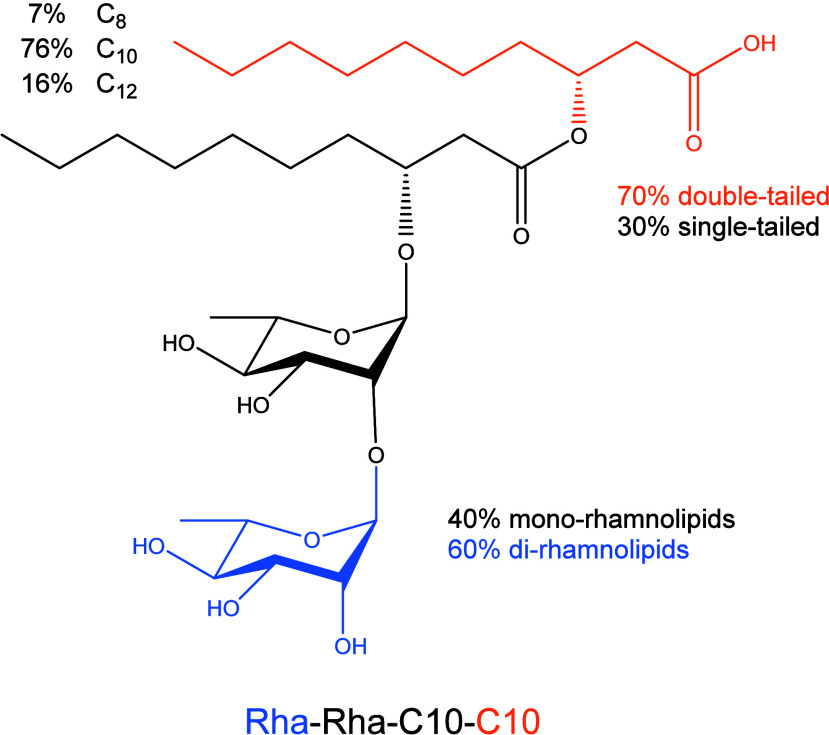
Molecular structures and relative amounts of rhamnolipid congeners present in the Rha sample investigated in this study. Congener composition was obtained as explained in Section 3.1.

One rhamnose unit is linked to the first lipid via an O-glycosidic bond. The fatty acid chains may differ both in length, typically ranging from C8 to C24, and in the degree of unsaturation. The lipids are linked by an ester bond formed between the carboxyl group of the first chain and the β-hydroxy group of the second chain, if present. The carboxyl group of the second chain remains typically unbound. This molecular structure prompts two considerations:

1. First, the presence of multiple hydrophilic moieties (the rhamnose(s) plus a carboxyl group) and the tail(s) arranged in a composite architecture^10,11^ is observed. Consequently, rhamnolipid self-aggregation behavior in water cannot be easily rationalized.
2. Second, all commercially available rhamnolipid batches present a marked chemodiversity, being mixtures of congeners, with more than 60 structurally distinct components identified.^12,13^ The composition of these mixtures is influenced by both the microbial strain and the cultivation conditions.^14,15^ Researchers have noted that to accurately identify the molecular determinants of rhamnolipid self-aggregation behavior, it is essential to study purified or synthetically obtained samples containing a single congener.^10,16^

The coexistence of various congeners is often identified as a limitation that may hinder the full exploitation of rhamnolipids in industrial formulations. However, a more thorough examination reveals that this diversity does not inherently constitute a problem. The molecular polydispersity of widely used synthetic surfactants is increasingly recognized as a pivotal factor in tuning their self-aggregation behavior (see, e.g., recent results for alkyl ethoxylates^17^ and alkyl ethoxysulfates^18^). The structural and functional features of a commercial sample of rhamnolipids depend on the specific combination of different congeners present in its composition and the nature of their intermolecular interactions. As long as the production of a rhamnolipid mixture can be standardized and its composition can be analyzed and reliably reproduced, investigating its structural and dynamic features and interpreting them from molecular to macroscopic length scales provide the basis for its rational use in formulation technology.

In this context, the present study explores the structure and flow behavior of a representative commercial rhamnolipid sample (hereafter referred to as Rha) in aqueous mixtures, extending the investigation to highly concentrated systems (up to 90 wt %). The work is divided into two closely related parts. In the first part, by combining gas chromatography–mass spectrometry (GC−) and high-performance liquid chromatography–mass spectrometry (HPLC-MS) with nuclear magnetic resonance (NMR) spectroscopy, the composition of the Rha sample is analyzed. This includes a detailed examination of the distribution of rhamnolipid congeners and other components present in the commercial sample. The second part of the study uses this information to identify the molecular determinants of the structural, rheological, and functional properties of Rha aqueous mixtures. By integration of visual inspection, polarized optical microscopy (POM), small-angle X-ray scattering (SAXS), and electron paramagnetic resonance (EPR), the aggregation behavior of Rha aqueous mixtures is investigated at different length scales, from the molecular organization to the aggregate morphology. Rheological measurements taken at different concentrations and temperatures reveals how these structural features influence the flow properties. Finally, the cleaning performance of Rha aqueous mixtures is tested.

Therefore, this work aims to develop solid correlations between congener composition and the structural and functional properties of a commercial sample of rhamnolipids, ultimately laying the scientific and technological foundations for their use in sustainable ultraconcentrated formulations.

## 2 Experimental Section

### 2.1 Materials

A commercial rhamnolipid mixture from the strain *P. aeruginosa* (Rha, 85–90% pure in solid/granular form and brownish in color) was purchased from AGAE Technologies (AGAE Technologies, LLC, Corvallis, Oregon, USA). All of the other chemicals, whose list is reported in the Supporting Information, were of analytical grade. Unless otherwise stated, ultrapure deionized water from a Millipore Milli-Q system with an electrical conductivity of less than 1 × 10^–6^ S cm^–1^ at 25 °C was used as the solvent.

### 2.2 Analysis of the Rha Composition

NMR experiments were performed on samples prepared as 10% w/w solutions in D_2_O. All spectra were acquired on a Bruker Avance 600 MHz (Rheinstetten, Germany) spectrometer equipped with a 5 mm triple resonance ^1^H­(^13^C/^15^N), *z*-axis pulsed-field gradient probe head. 1D ^1^H and ^13^C NMR spectroscopy, 2D ^1^H–^1^H correlation spectroscopy (COSY), ^1^H–^13^C heteronuclear single quantum coherence (HSQC), HSQC-total correlation spectroscopy (TOCSY) experiments, ^1^H–^13^C heteronuclear multiple bond correlation (HMBC) spectroscopy, and pseudo-2D diffusion-ordered spectroscopy (DOSY) experiments were run using the settings specified in the Supporting Information. All spectra were processed by using Bruker TopSpin 4.4.0 and MestreNova 9 (MestreLab Research S.L., Santiago de Compostela, Spain).

The LC-MS analysis of the Rha sample was performed using an Agilent LC-MS electrospray ionization time-of-flight (ESI-TOF) 1260/6230DA (Cernusco sul Naviglio, Milan, Italy) instrument operating in negative ionization mode and interfaced to an Agilent Eclipse Plus ODS column. The Supporting Information reports details of the extraction procedure, eluents, column, and instrumental settings.

The GC-MS analyses were performed according to two different sample preparation protocols: In experiment 1, an aliquot of Rha was hydrolyzed in 2 M HCl and extracted with EtOAc. In experiment 2, a weighted amount of Rha was dissolved in water, acidified to pH ∼ 2, and extracted with EtOAc. In both protocols, organic phases were dried on Na_2_SO_4_ and evaporated under reduced pressure. The residue was derivatized with BSTFA and analyzed by GC-MS using an Agilent 6850 GC (Cernusco sul Naviglio, Milan, Italy), equipped with an HP-5MS capillary column (5% phenyl methyl polysiloxane stationary phase), coupled to an Agilent 5973 Inert MS detector operated in full-scan mode. A detailed description of the experimental setup, including column temperature and MS scanner frequency, is reported in the Supporting Information. The identification of fatty acids was performed by matching their EI mass spectra at 70 eV with those stored in the NIST 20 mass spectral library (https://www.nist.gov/srd/nist-standard-reference-database-1a) and supported by the Kovats retention index (RI) calculated for each metabolite by the Kovats equation using the standard *n*-alkane mixture in the range C7–C40 (Sigma-Aldrich, Saint Louis, Missouri, USA) analyzed under the same conditions. Palmitic acid (chromatographic peak at about 13.5 min) was selected as a housekeeping internal standard.

### 2.3 Analysis of the Aggregation and Flow Behavior of Water–Rha Mixtures

Samples for POM, SAXS, EPR, and rheology measurements were prepared by weighing appropriate amounts of Rha and Millipore deionized water to obtain mixtures with weight-to-weight percentages ranging from 20 to 90%. Details of the preparation and equilibration procedure are reported in the Supporting Information. The pH of the diluted samples (less than 10 wt % Rha) was measured to be around 6, which is close to the p*K*_a_ of the carboxylic group of the self-aggregated rhamnolipids.^11^ Since high concentrations depress weak acid dissociation, the rhamnolipids were considered to be predominantly in their undissociated form.

A detailed description of sample loading, instrumental settings, and data treatment for each kind of measurement is reported in the Supporting Information. Briefly, POM images were collected using an Axiovert 200 M or a Cell Observer light microscope (Carl Zeiss Light Microscopy, Germany) and a homemade incubator capable of maintaining a constant sample temperature within 0.1 °C. Observations were made between crossed polarizers.^19^ Representative images were recorded using an AxioCam HRm high-resolution digital camera.

SAXS experiments were conducted at the Diamond Light Source B21 Beamline (Didcot, United Kingdom). SAXS data were collected in the temperature range 20–50 °C with 10 °C temperature increase steps. Further details on the experimental settings can be found in the literature.^20^ Experimental data of the samples containing up to 50 wt % Rha were fitted using the SASView v5.0.6 Software (www.sasview.org) using a custom module made of the combination of the Power Law and the Core–Shell Ellipsoid models.^21^ EPR spectra of the spin probe 16-DOXYL-stearic (16-DSA) in water–Rha mixtures were acquired at room temperature (25 ± 2 °C) by using a Bruker Elexsys E-500 X-band spectrometer (9.87 GHz, Rheinstetten, Germany). The spectra were simulated using the computational approach described by Budil et al.^22,23^ The rheological properties of the system were characterized using an MCR302 evolution stress-controlled rheometer (Anton Paar GmbH, Graz, Austria), equipped with Taylor-Couette (concentric cylinder) geometry.

### 2.4 Cleaning Efficiency Test

Cleaning efficiency was assessed using a simplified procedure based on recommendations from IKW (Industrieverband Körperpflege and Waschmittel), the German Cosmetics, Toiletries, Perfumes and Detergents Association, for cleaning performance.^24^ This method measures the amount of oil, quantified as weight loss%, that the surfactant mixture can absorb from a surface,^25^ as detailed in the Supporting Information.

## 3 Results and Discussion

### 3.1 Analysis of the Rha Composition

#### 3.1.1 NMR Spectroscopy Results

NMR provides the opportunity to determine the molecular structure and relative amounts of the different compounds present in the Rha sample simultaneously, without any preparation apart from dissolution in deuterated water, and without the need for pure rhamnolipid congeners as reference standard.^26^ NMR enables the investigation of not only the Rha congener distribution but also the potential presence of other compounds, such as other bacterial metabolites, in the sample. Two-dimensional ^1^H–^13^C HSQC-TOCSY, HMBC, and pseudo-2D DOSY experiments were performed to facilitate the unambiguous assignments of peaks of specific rhamnolipid signals. In particular, the region between 4.8–5.0 ppm (^1^H) and 90–105 ppm (^13^C) in the HSQC spectra displayed the signals corresponding to the anomeric carbons and protons of the rhamnose moiety from four distinct species, including two monorhamnolipids (single- and double-tailed) and two dirhamnolipids (single- and double-tailed, Figure 2A).

**Figure 2:**
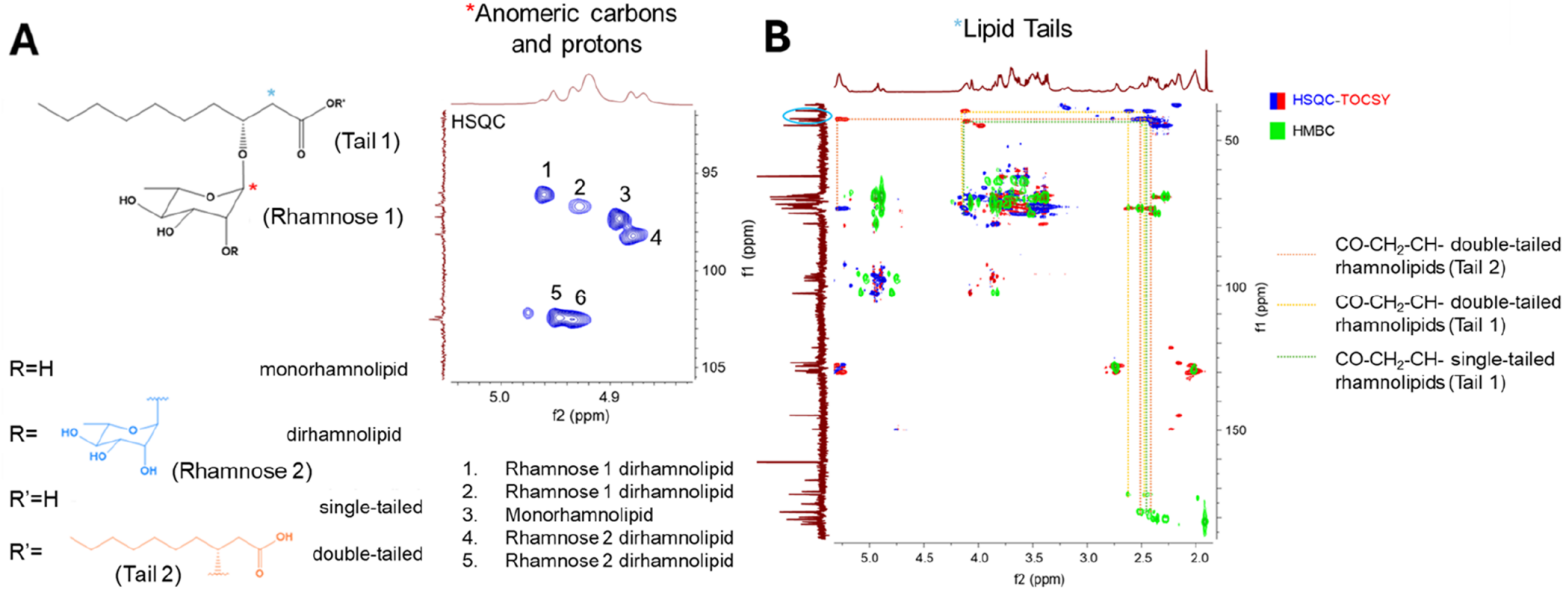
(A) Snapshot of the HSQC spectrum region between 4.8–5.0 ppm (^1^H) and 90–105 wdasppm (^13^C) including the signals corresponding to the anomeric carbons and protons of the rhamnose sugars. (B) Snapshot of the HSQC-TOCSY and HMBC spectra region between 2.0–5.5 ppm (^1^H) and 40–185 ppm (^13^C) including the correlations between the signals of the carboxyl groups, α-methylenes, and β-methyls in the lipid tails.

Additional diagnostic signals include the proton in the β-position relative to the carboxyl group on the lipid tail: In double-tailed rhamnolipids, it was possible to clearly distinguish between the proton on the first tail (adjacent to the rhamnose group) and that on the second tail. The proton on the first tail appears at 4.12 ppm, while the second resonates at 5.28 ppm, considering the deshielding effect of the two carboxyl groups. In the single-tailed rhamnolipid, instead, the analogous proton resonates at 4.11 ppm. Based on these characteristic chemical shifts and the observation of quaternary carbon correlations in the HMBC spectra, methylene signals in the α-position relative to the carboxyl groups from the lipid tails could be assigned, identifying two distinct methylene signals for double-tailed rhamnolipids and one for monotailed rhamnolipids (Figure 2B).

Pseudo-2D DOSY experiments further supported these assignments by discriminating rhamnolipid micelle signals based on their diffusion coefficients, which ranged between 5.6 × 10^–11^ and 5.05 × 10^–11^ m^2^/s. DOSY also enabled the detection of impurities such as formic acid and acetic acid as well as the identification of heavier species, consistent with other glycolipids, and lighter species, indicative of free fatty acids and free sugar units (Figure S1). The observed chemical shifts (Figure S2) were in agreement with previously reported data.^27–29^ This is an interesting case in which the DOSY experiment was an important step in analyzing the composition of complex mixtures using NMR,^30^ enabling the rapid screening of Rha components other than rhamnolipids.

The assignment of the anomeric carbon signals of the rhamnose heads and the methylene groups in the α-position relative to the carboxyl groups in the lipid tails enabled the identification and quantification of the rhamnolipid species within the mixture. Integration of the carbon signals from quantitative 1D ^13^C NMR (zgig30) revealed a composition of 60% dirhamnolipids vs 40% monorhamnolipids, and 70% double-tailed rhamnolipids vs 30% single-tailed rhamnolipids (Figure 3).

**Figure 3:**
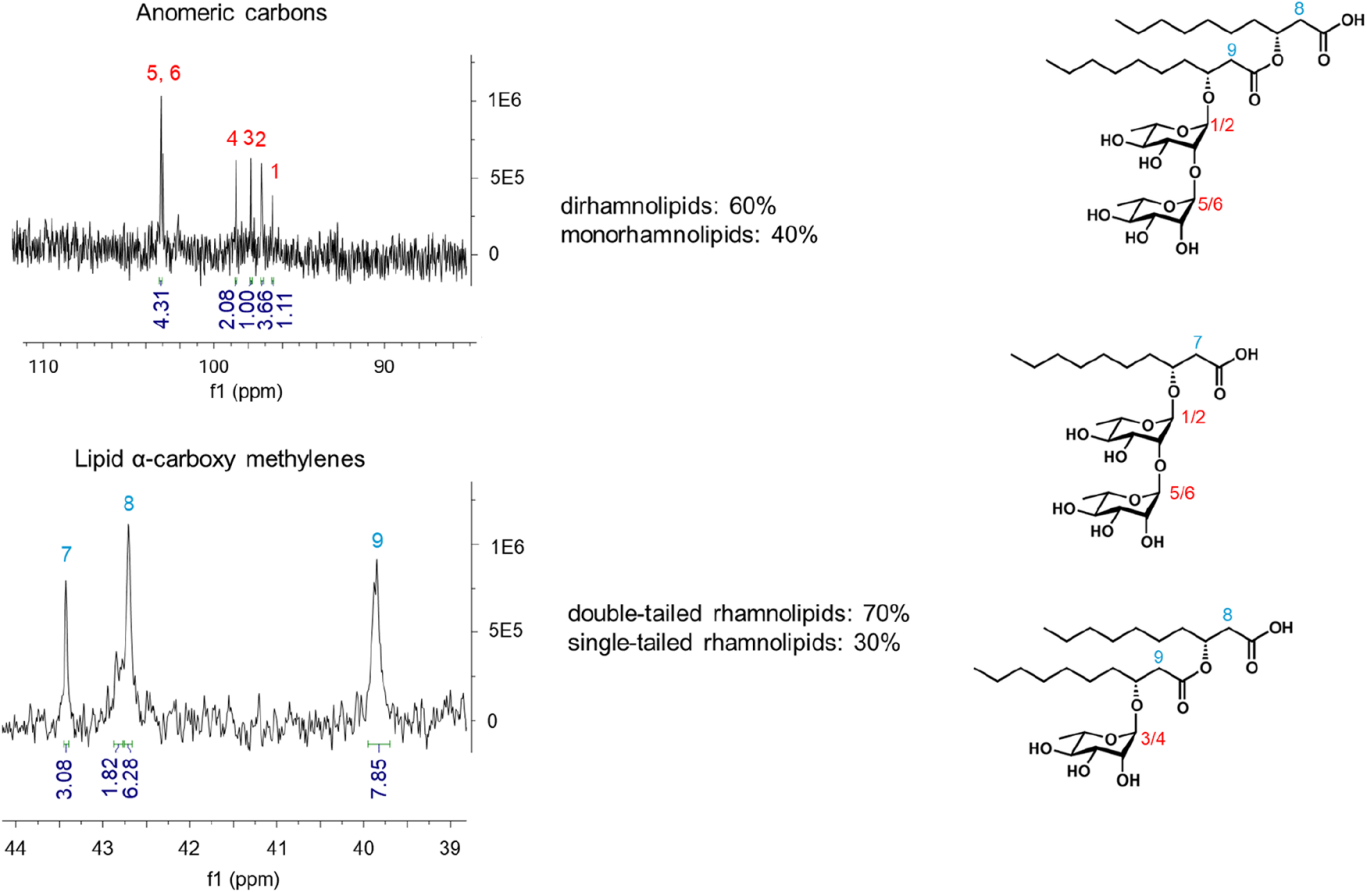
Integration of the quantitative 1D ^13^C spectrum in the regions of the rhamnose head anomeric carbons and lipid tail α-carboxy methylene carbons, with the corresponding positions indicated in the 2D representations on the right.

The quoted percentages are mol % because the integral of the peaks is proportional to the number of resonant nuclei. These NMR results align with literature data, obtained by chromatographic techniques, showing a prevalence of dirhamnolipids in the rhamnolipid mixtures obtained by *P. aeruginosa* using glycerol/sugars as the carbon source.^13,27–29^

#### 3.1.2 HPLC-ESI-MS and GC-MS Results

Chromatographic techniques coupled with mass spectrometry are the methods of choice for rhamnolipid analysis,^31^ since these methods allow the precise identification of individual congeners (HPLC-MS) and fatty acid length, unsaturation and branching (GC-MS). However, these methods require effective sample preparation protocols to remove interfering compounds prior to the analysis.^13,32^ Moreover, accurate quantification still has some drawbacks^33^ as it requires suitable pure rhamnolipid congeners as standards to be available.

The molecular structures of the rhamnolipids present in the Rha sample were obtained by HPLC-ESI-MS analysis. The Rha mixture was dissolved in acidified water at a pH ∼ 2. This pH value is low enough to prevent dissociation of the carboxylic group of the rhamnolipids, thus facilitating extraction in EtOAc. It is also high enough to prevent rhamnolipid hydrolysis. Table 1 shows the rhamnolipids present in the EtOAc extract.

**Table 1:** Rhamnolipids Present in the Rha Sample Detected by HPLC-ESI-MS Analysis^a^

compound	molecular formula	molecular weight (Da)	pseudomolecular ion [M–H]^−^ (*m*/*z*)
Rha-C8	C_14_H_26_O_7_	306	305.16
Rha-C10	C_16_H_30_O_7_	334	333.20
Rha-C12-CH3^b^	C_19_H_36_O_7_	376	375.27
Rha-C10-C8	C_24_H_44_O_9_	476	475.29
Rha-C10-C10	C_26_H_48_O_9_	504	503.15
Rha-Rha-C8	C_20_H_36_O_11_	452	451.30
Rha-Rha-C10	C_22_H_40_O_11_	480	479.25
Rha-Rha-C12	C_24_H_44_O_11_	508	507.28
Rha-Rha-C8-C10	C_30_H_54_O_13_	622	621.34
Rha-Rha-C10-C10	C_32_H_58_O_13_	651	649.98
Rha-Rha-C10-C12:1	C_34_H_60_O_13_	676	675.39

a In the case of double-tailed Rha, the order for tails is not assigned.^31^

b Rha-10-methyl-3-hydroxy-dodecanoic acid.

To gather more detailed information on the fatty acid composition of the Rha sample used in this study, a GC-MS analysis was performed. Two types of integrated experiments were performed, referred to as experiments 1 and experiment 2.

Experiment 1 was aimed at identifying all of the fatty acids present in the sample, both in free form and bound to rhamnose in rhamnolipids. To this end, the mixture was exhaustively hydrolyzed to liberate the rhamnose-bound fatty acids, which were then extracted with EtOAc. Fourteen fatty acids were identified in the GC-MS chromatograms of the trimethylsilylated extract (Figure S3). In particular, five different 3-hydroxy fatty acids were detected comprising 3-hydroxy-hexanoic acid (C6-OH), 3-hydroxy-octanoic acid (C8-OH), 3-hydroxy-decanoic acid (C10-OH), 3-hydroxy-dodecanoic acid (C12-OH), and 10-methyl-3-hydroxy-dodecanoic acid (CH3-C12-OH).

The aim of experiment 2 was to identify only the free fatty acids in the mixture, excluding the hydroxy acids bound to rhamnose. Accordingly, the mixture was dissolved in acidified water and extracted with EtOAc. The resulting extract represented 22.5 wt % of the total fatty acid content, as determined in experiment 1. Comparison between the results of experiments 1 and 2 confirms that a fraction of each of the five 3-hydroxy fatty acids present in the sample is bonded to rhamnose (Figures S3 and S4). However, this fraction could be confidently evaluated quantitatively only for C8-OH, C10-OH, and C12-OH. Quantitative analysis of the results shows that, with respect to the total lipids bound to rhamnose, the percentages of 3-hydroxy fatty acids are ∼7.3% C8-OH, ∼76.2% C10-OH, and ∼16.5% C12-OH. Thus, the predominant rhamnolipids in the mixture include 3-hydroxydecanoic acid. Those containing 3-hydroxydodecanoic and 3-hydroxyoctanoic acids are present at substantially lower levels. These results are in agreement with the literature showing a strong prevalence of C10 tails in rhamnolipid mixtures.^27–29^

The rich chemodiversity of the Rha sample deserves some comments. First of all, the content of free fatty acids in Rha is quite high, as evidenced by both NMR and GC-MS analysis, and already reported in literature.^34^ Free fatty acids can act as cosurfactants, playing a relevant role in self-aggregating systems.^35^ Second, unsaturated fatty acids may significantly impact the packing of surfactants and lipids in supramolecular aggregates.^36^ Furthermore, they can undergo oxidation processes that influence the system reactivity. Finally, although the presence of branched fatty acids is very minor, it should not be neglected. Some recent studies have reported the presence of short branches, often methyl, near the terminals of acyl chains in biosurfactants^37^ and other amphiphilic bacterial molecules, such as lipopolysaccharides.^38^ While their role remains to be determined, it is important to note that the presence of a branch can significantly impact the self-aggregation process of surfactants.^4^

### 3.2 Analysis of the Aggregation and Flow Behavior of Water–Rha Mixtures

#### 3.2.1 Polarized Optical Microscopy

The phase behavior of the water–Rha system at 25 °C was preliminarily analyzed by visual inspection and POM. The photographs of the selected samples taken a few seconds after they were tilted are shown in Figure 4 and Figure S5.

**Figure 4:**
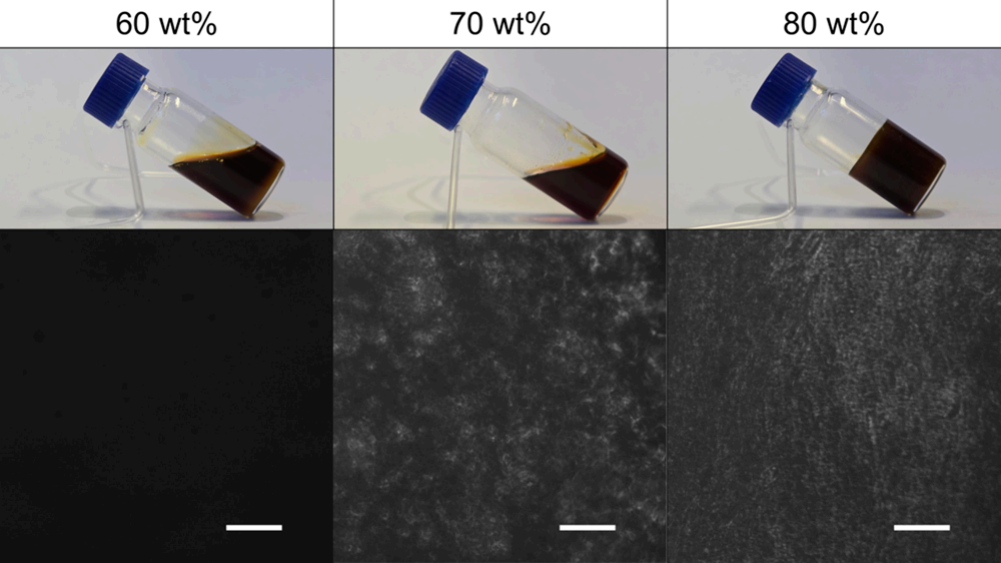
Visual and POM analyses of selected water–Rha mixtures at 25 °C. The top images are photographs of the samples taken 5 s after tilting. The bottom images display representative POM images under crossed polarizers. The scale bar is 100 μm.

Samples with a concentration of up to 75 wt % flow under their own weight. Conversely, those with a composition of 80 wt % or higher do not flow, exhibiting the consistency of hard gels. Rha does not completely dissolve at a concentration of 90 wt %. Figure 4 also shows micrographs of the same samples acquired using crossed polarizers. Samples with a concentration of up to 60 wt % do not exhibit birefringency. This indicates the formation of an isotropic L1 phase. Conversely, samples with a composition of 70 wt % or higher are birefringent. This evidence indicates the formation of optically anisotropic lyotropic liquid crystalline (LLC) structures, such as hexagonal or lamellar ones. However, a thorough examination of the POM images does not reveal any texture characteristics indicative of specific structural features, such as the presence of maltese crosses or fan-like structures for lamellar and hexagonal LLCs, respectively. The effect of temperature was also analyzed (up to 50 °C), and no significant changes were observed. İkizler et al. found similar results when they analyzed mixtures of mono- and dirhamnolipids at various molar ratios.^39^ They observed a mosaic of small, different birefringent domains indicative of a complex polymorphism. On the other hand, maltese crosses were clearly visible in samples of individual congeners.^40^ Thus, the POM images in Figure 4 demonstrate how the coexistence of various congeners in the Rha sample influences aggregation behavior.

#### 3.2.2 Small-Angle X-ray Scattering

To better analyze the structural changes occurring in water–Rha mixtures as the concentration increases, an SAXS investigation was undertaken. The experimental SAXS 1D profiles of the samples containing 10–50 wt % Rha, along with the fitting curves, are shown in Figure 5. By applying an ellipsoidal core–shell model, it was possible to fit the higher-*q* region, thereby extracting the structural parameters of the Rha aggregates reported in Table 2. In the low-*q* region, for samples with Rha content equal to or higher than 30 wt %, the scattered intensity exhibited a characteristic power law decay proportional to *q* ^–3^ indicative of mass fractals,^41,42^ which could be interpreted as due to coexisting large-scale structures.^16^

**Figure 5:**
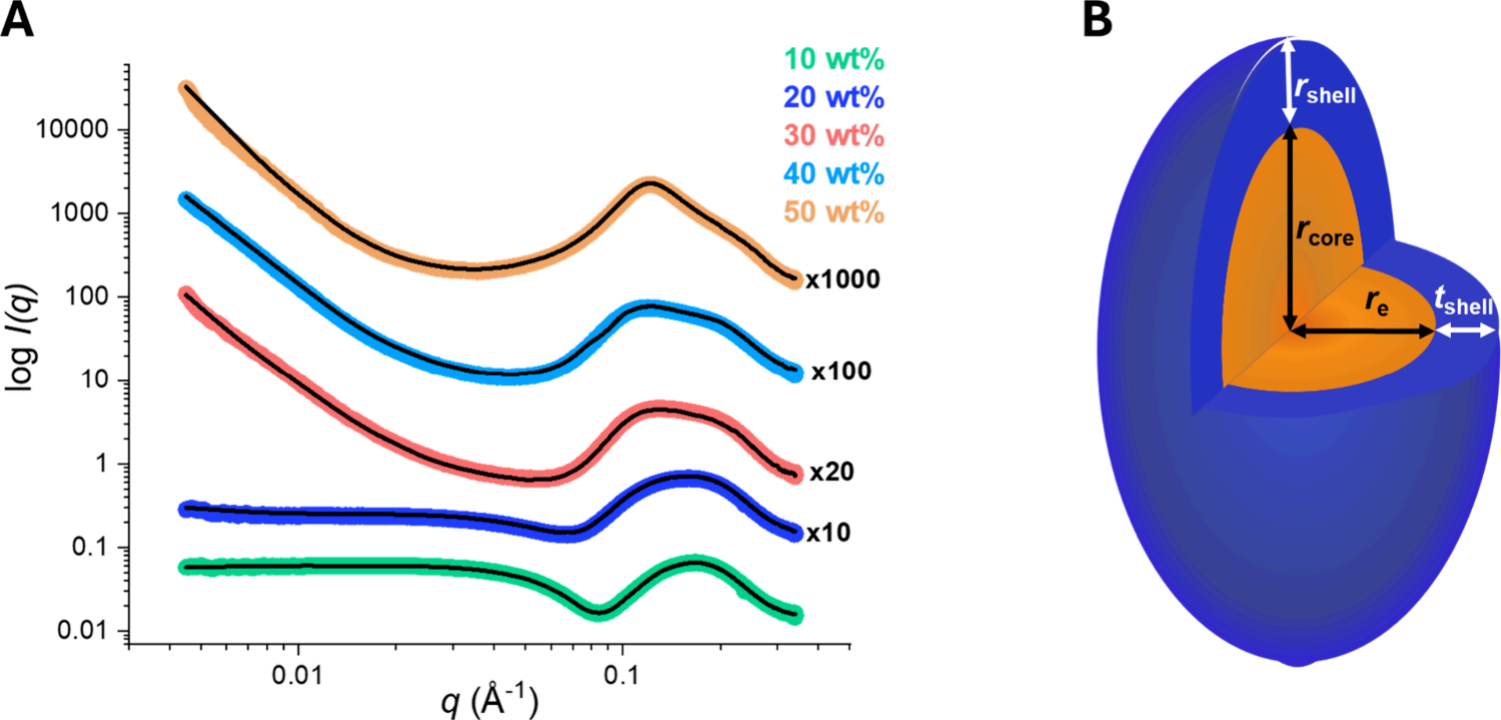
(A) SAXS profiles of water–Rha mixtures at concentrations ranging from 10 to 50 wt % at 20 °C (colored lines). Model fits to the experimental data are shown as black lines. (B) Schematic 3D representation of the aggregate morphology.

**Table 2:** Structural Parameters of Ellipsoidal Aggregates Obtained from Fitting of the SAXS Data at 20 °C^a^

concentration	*r*_core_/*r*_e_	*t*_shell_/*r*_shell_	*t*_shell_ (Å)	*r*_e_ (Å)
10 wt % Rha	4.1	1.1	14.9 ± 0.5	11.0 ± 0.5
20 wt % Rha	3.9	1.1	15.1 ± 1.0	10.8 ± 0.5
30 wt % Rha	4.3	1.1	13.5 ± 1.0	10.0 ± 0.5
40 wt % Rha	3.9	1.1	15.0 ± 1.0	10.1 ± 0.5
50 wt % Rha	3.9	1.1	15.1 ± 1.0	10.1 ± 0.7

a *t*_shell_, *r*_shell_, *r*_e_, and *r*_core_ are the equatorial and polar shell thicknesses and the equatorial and polar core radii, respectively.

Inspection of Table 2 reveals that the structural parameters remain nearly constant when the Rha content of the mixtures is changed, indicating the formation of prolate micelles with an aspect ratio *R*_p_/*R*_e_ = (*r*_core_ + *r*_shell_)/(*r*_e_ + *t*_shell_) of about 2.2, where *R*_p_ and *R*_e_ are the polar and equatorial radii of the aggregates, respectively. These aggregates are composed of an elongated hydrophobic core surrounded by a uniform shell of Rha hydrophilic heads, approximately 15 Å thick. The equatorial radius of the core, *r*_e_, is consistent with the extended conformation of the tails of the prevalent rhamnolipid congeners present in the mixture (∼10 Å).^43^ The independence of micelle size and shape from concentration is relatively uncommon, as an increase has been observed for other surfactants.^44^ A similar behavior has already been reported for other glycosylated surfactants^45^ and may be related to the repulsion among the bulky headgroups, which limits micelle growth.

Interestingly, the aspect ratio observed in the present work for the Rha mixture (∼2.2) is close to those reported for the pure Rha-Rha-C10-C10 (1.9) while it differs significantly from the aspect ratio of Rha-C10 micelles (6.2).^16^ This leads to the conclusion that the aggregate morphology is ruled by the congeners present in higher amount, which are double-tailed dirhamnolipids. A confirmation of this conclusion comes from the evidence that mixtures enriched in monorhamnolipid double-tailed congeners, such as the sample studied by Haba et al., were found to form bilayer domains, as revealed by SAXS profiles.^46^ This behavior closely mirrors the aggregation observed in systems composed of pure monorhamnolipid double-tailed specie, Rha-C10-C10, which have been shown by Baccile et al. and Marega Motta et al.^16,47^ A closer inspection of the results prompts important consideration. In fact, the equatorial radius of the Rha micelles (∼25 Å) is significantly larger than that found for the pure congeners (∼20 Å). The formation of larger aggregates may indicate the inclusion of free fatty acids, which act as cosurfactants.^48^

SAXS profiles obtained for samples with Rha content equal or above 60 wt % are shown in Figure 6 and Figure S6.

**Figure 6:**
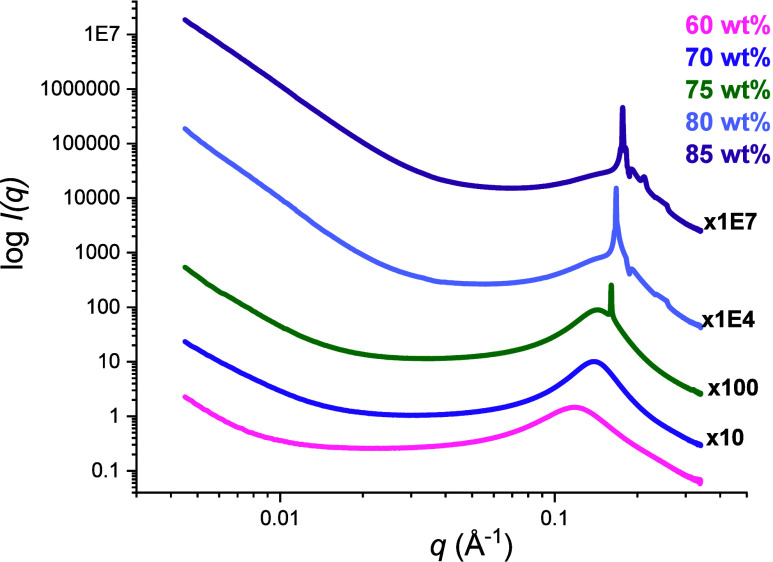
SAXS profiles of water–Rha mixtures at concentrations ranging from 60 to 85 wt % at 20 °C.

Interestingly, the models used so far failed to satisfactorily fit the SAXS profiles obtained for the samples at 60 and 70 wt %. When the Rha content was 75 wt %, the SAXS patterns exhibited a sharp peak that became more pronounced with increasing concentration. Additional minor peaks were also detected for the samples at 80 and 85 wt % (see Table S1). This evidence points to the formation of either LLC domains or solid crystals. However, all attempts to univocally identify a single-crystalline structure were unsuccessful. Recent studies report that, for the pure dirhamnolipid, the formation of a lamellar phase occurs,^7^ while single-tailed monorhamnolipids form hexagonal phases.^8^ Our findings suggest that the presence of different congeners and free fatty acids could lead to a progressive structural reorganization of the mixtures, possibly forming multiple, yet not well-ordered, Rha supramolecular assemblies that can coexist. From the position of the predominant peak, it was possible to calculate the corresponding correlation distance (Table 3), representing the average separation between aggregates.

**Table 3:** Correlation Distance Obtained from the SAXS Profiles of the Concentrated Water–Rha Mixtures at 20 °C

concentration	*q* (Å^–1^)	correlation distance (Å)
70 wt % Rha	0.1385	45
75 wt % Rha	0.1463	44
80 wt % Rha	0.1695	37
85 wt % Rha	0.1776	35

The correlation distances decreased with increasing Rha concentration, indicating a reduction in the average interaggregate spacing. This behavior underscores the transition toward a more densely packed system, where the aggregates are forced into closer proximity as a result of concentration-induced crowding effects.

The same water–Rha mixtures were investigated by SAXS at temperatures increased in steps of 10 up to 50 °C. All samples exhibited minimal temperature dependence (Table S2). In particular, the core–shell ellipsoidal aggregate structures remained unchanged for samples containing 10–50 wt % Rha. Moreover, in the case of higher-concentration samples, with a range of 75–85 wt % Rha, there is negligible alteration in the position of the sharp peaks. The scarce dependence of the aggregation behavior on temperature is a common feature of sugar-based surfactants and is due to the strength of the hydrogen bonds between the hydroxyl groups of the sugar units and water. This prevents any significant dehydration of the headgroup as the temperature increases.^49^

#### 3.2.3 Electron Paramagnetic Resonance

EPR spectroscopy was used to study the local polarity and microviscosity experienced by amphiphiles in water–Rha mixtures at concentrations ranging from 10 to 85 wt %. The spin probe 16-DSA was selected because it bears the reporter group (the doxycyclic nitroxide) in close proximity to the terminus of the saturated fatty acid chain. This positioning is optimal for effective embedding within the inner part of the hydrophobic core of aggregates formed by surfactants and/or lipids.^50^ The spectra of 16-DSA incorporated in water–Rha mixtures at increasing Rha contents are shown in Figure 7.

**Figure 7:**
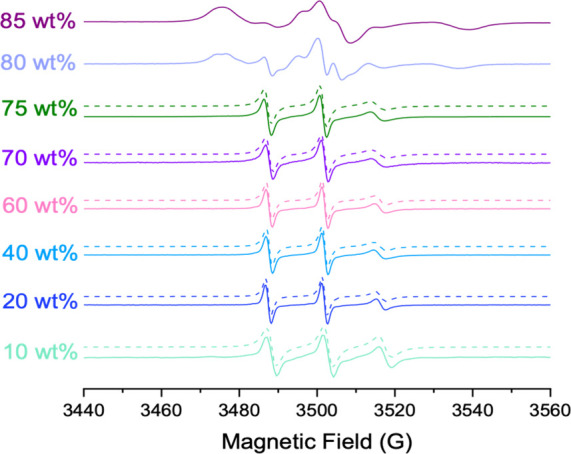
Experimental (solid lines) and simulated (dashed lines) EPR spectra of 16-DSA in water–Rha mixtures at 25 °C.

For Rha contents up to 75 wt %, the spectra are characteristic of fast-moving nitroxides in a liquid at an oily viscosity, only showing the three hyperfine lines due to the coupling between the unpaired electron and the ^14^N nitrogen nucleus (*I* = 1). The three peaks become broader with increasing Rha content, evidence of an increasingly hindered motion of the paramagnetic probe, which is thus embedded in a more viscous microenvironment. No evidence of spectral anisotropy is detectable indicating the rotation along all directions to be not distinguishable.^51^ This evidence points to a rather compact but disordered inner core of Rha micelles. At 80 and 85 wt % Rha, the 16-DSA spectra show marked deviations: An evident overlap of multiple components is observed, signaling increased structural heterogeneity and reduced probe mobility.

A thorough analysis of the EPR spectra was achieved by simulating them using the computational approach described by Budil et al.^22^ For the spectra obtained at Rha content of 75 wt % or less, a satisfactory agreement between experimental and computed spectra was attained by assuming a single component in the system, which presents the spectral features of radical with fast motion. This means that all the 16-DSA molecules embedded in a similar microenvironment are solubilized in the Rha micelles. Simulating these spectra provided values for the average hyperfine coupling constant, ⟨*A*⟩ = (*A*_*xx*_ + *A*_*yy*_ + *A*_*zz*_)/3, and the rotational correlation time (τ_c_). ⟨*A*⟩ and τ_c_ are considered as indexes of the polarity and microviscosity of the local environment in which the reporter group is embedded, respectively. Inspection of Figure 8 shows that the values of the polarity parameter ⟨*A*⟩ decrease slightly from 14.55 to 14.25 G.

**Figure 8:**
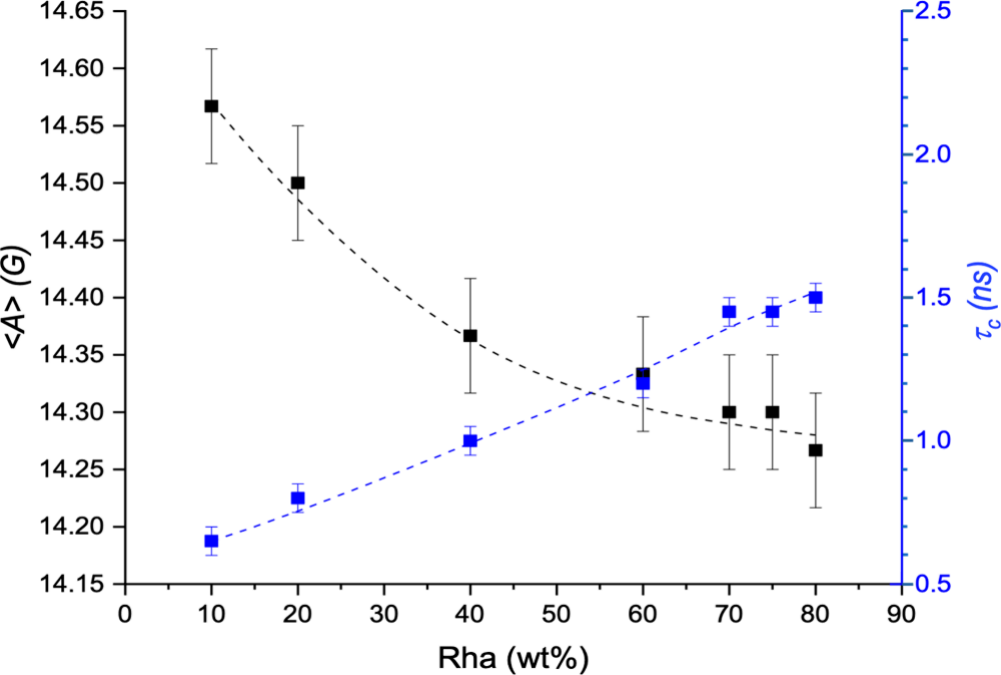
Polarity parameter ⟨*A*⟩ (left axis) and rotational correlation time τ_c_ (right axis), obtained from the simulation of the fast-motion spectra of 16-DSA at 25 °C, reported as a function of the Rha concentration in the aqueous mixture.

These values are indicative of a low-dielectric microenvironment, ranging between those experienced by 16-DSA in long-chain alcohols (⟨*A*⟩ = 14.8 G in heptanol^51^ and alkanes (⟨*A*⟩ = 14.2 G in dodecane^51^), thus demonstrating that the reporter group is deeply embedded in the hydrophobic micellar core. Concurrently, the rotational correlation time τ_c_ increases with increasing Rha content, reflecting a gradual restriction of probe mobility within increasingly viscous domains. Interestingly, the ⟨*A*⟩ values found in this work are significantly lower, while the τ_c_ values are higher than those reported for micelles formed by pure monorhamnolipids^43^ or other surfactants.^52,53^ This evidence could be attributed to the increased bulkiness of the aggregates due to the solubilization of long-chain free fatty acids as cosurfactants, which creates a microenvironment in the inner core that is less permeable to water molecules and more viscous. Another possible contribution comes from branched fatty acids, which have been shown to form tightly assembled structures, the short side chains occupying the voids between the linear ones.^4^

The simulation of the 16-DSA spectrum in 80 wt % Rha reveals, in addition to a fast-motion component similar to that found at lower Rha concentration, the emergence of a slow-motion component, as shown in Figure 9. This evidence indicates the partitioning of the probe between two different environments, one of which is similar to those found in Rha micelles while the other is significantly more viscous (τ_c_ = 16.7 ns). The ⟨*A*⟩ value obtained for this second component (∼16 G) indicates a more polar environment and suggests that the DSA chain is folding to expose the terminus to the aqueous medium (⟨*A*⟩ = 15.8 G in water^51^). Notably, this slow component could be accurately simulated without invoking an order parameter, indicating that the spin probes are immobilized without the need for orientational bias.

**Figure 9:**
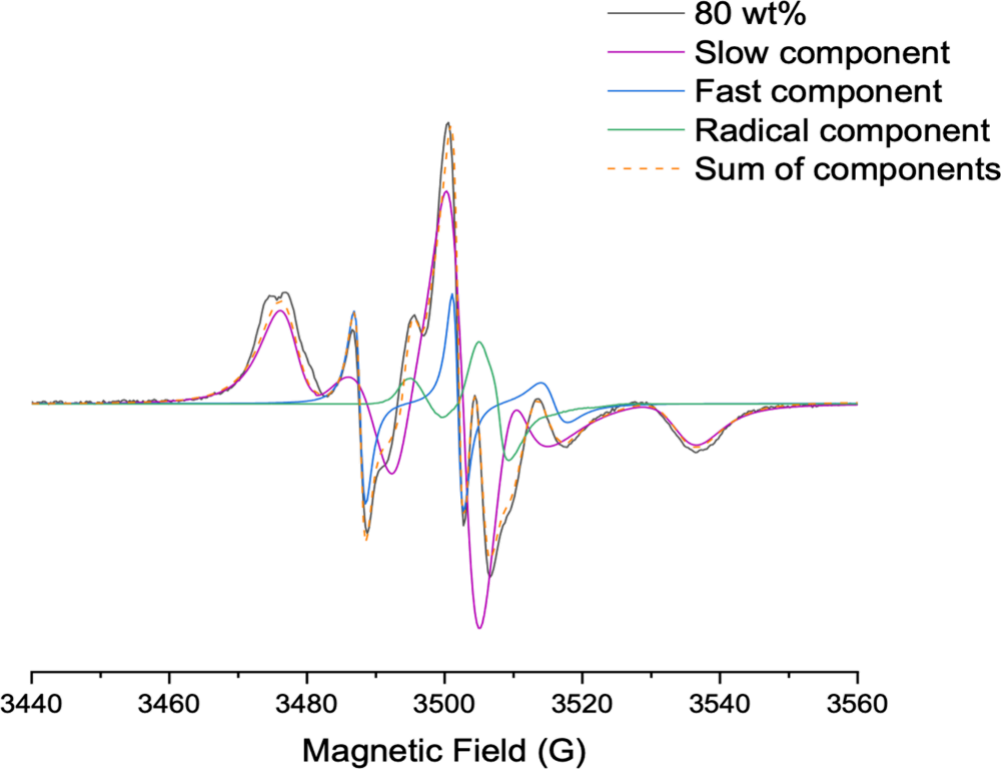
Experimental (solid line) and simulated (dashed line) EPR spectra of 16-DSA in the water–Rha mixture at 80 wt % and 25 °C.

Residual analysis following subtraction of the fast- and slow-motion components from the experimental spectrum unveiled two minor signals not directly due to the 16-DSA nitroxide group but rather ascribable to endogenous carbon-centered radicals, likely originating from high local concentrations and light-triggered processes. These signals, centered at ⟨*g*⟩ = 2.0020 ± 0.0002 and 2.0035 ± 0.0002, could be related to partial oxidations of either rhamnose^54^ or unsaturated lipid chains.^55^ Further investigation is needed to better ascertain the molecular origin of this finding. In all cases, it highlights the ability of concentrated Rha mixtures to stabilize radical species, which could be connected to the antioxidant properties of rhamnolipids demonstrated by various authors.^56^

A satisfactory simulation of the experimental spectrum at 80 wt % Rha was obtained by summing 70% of the slow-motion component, 15% of the fast-motion component, and 15% of the radical component (see Figure 9). The 16-DSA spectrum in 85 wt % Rha is similar to that obtained at 80 wt %. However, the slow component becomes strongly predominant, thus hampering a correct simulation of the other components.

#### 3.2.4 Rheology

Flow curves of water–Rha mixtures were recorded at concentrations between 10 and 80 wt % at temperatures of 15, 25, 37, and 45 °C. Figure 10A shows the flow curves at 25 °C.

**Figure 10:**
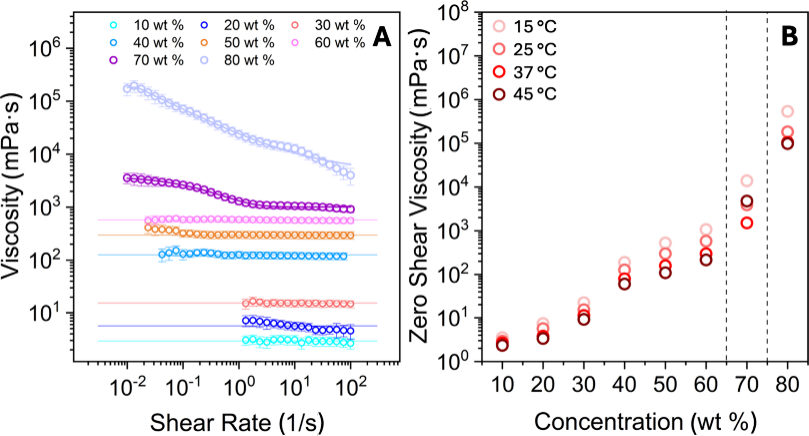
(A) Flow curves for water–Rha mixtures from 10 to 80 wt % at 25 °C. (B) Zero-shear viscosities at all concentrations and temperatures investigated. The linear fits in (A) represent Newtonian-like behavior, while the other behaviors were fitted with the Carreau–Yasuda model. The dotted lines in (B) indicate the onset of viscoelastic behavior.

Newtonian-like behavior was observed for concentrations below 70 wt %. Xiao et al. demonstrated that rhamnolipid viscosity increases with concentration in the 3–30 wt % range; our study extends this observation to much higher concentrations.^57^ A linear fit with a slope of zero was applied to these data. Conversely, apparent pseudoplastic behavior was observed at 70 and 80 wt %. This clearly indicates a phase transition. The Carreau–Yasuda model was used under these conditions to determine the zero-shear viscosity.^58^ The observed shear thinning behavior is typical of both hexagonal and lamellar LLCs^4^ and in systems with densely packed micelles,^59^ where alignment under shear reduces viscosity. Thus, these viscosity data are consistent with the presence of ordered structures. The zero-shear viscosities were plotted as a function of the Rha concentration at the investigated temperatures (Figure 10B). Clearly, a stable isotropic micellar phase exists up to 70 wt %, the limit beyond which the structural transition occurs.

To examine the system’s behavior during the transition more closely, oscillatory experiments were performed at concentrations of 70 and 80 wt %, as shown in Figure 11A. The frequency sweep at 70 wt % reveals a *G*″ (loss modulus) higher than *G*′ (storage modulus), while at 80 wt %, a viscoelastic behavior is observed with *G*′ exceeding *G*″ at low angular frequencies. Figure 11A shows the data at 45 °C; similar behaviors were recorded at all temperatures. Particularly, the 80 wt % sample exhibits viscoelastic behavior at all investigated temperatures, indicating an almost constant relaxation time with temperature, further confirming that the structure of the mixture does not change with temperature, as indicated by SAXS.

**Figure 11:**
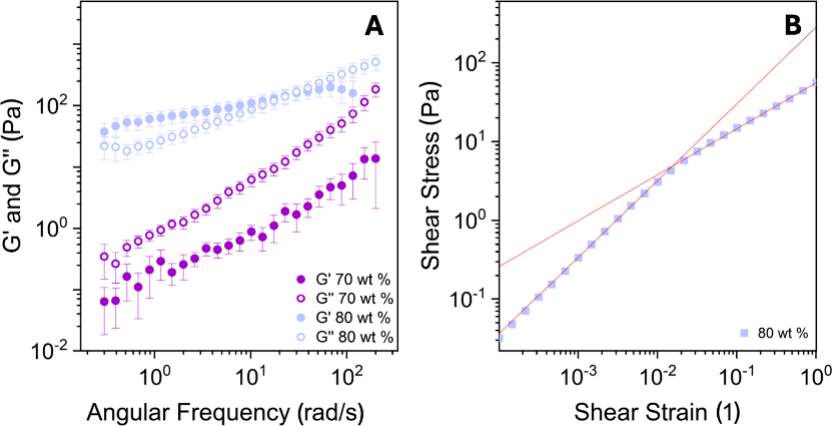
(A) Frequency sweep for water–Rha mixtures at 45 °C at 70 and 80 wt %. (B) Stress–strain profile obtained for the amplitude sweep experiments at 80 wt % and 25 °C.

These observations suggest that aggregate or network formation begins at 70 wt % but does not fully percolate through the system. The viscoelastic transition is clearly visible at 80 wt %. The sample at 80 wt % exhibits a yield stress (Figure 11B), implying that the previous analysis in Figure 10B should be considered indicative rather than rigorous for those concentrations, reflecting the viscosity at low shear rates.

### 3.3 Cleaning Efficiency Performance

The cleaning efficiency of Rha was tested, and the result was compared with those obtained for two commercial surfactants. The same surfactant content was considered (6.4 wt %). It was chosen in such a way that for all compared surfactants, the aqueous mixture is an isotropic micellar solution. Contaminated stainless-steel tiles were immersed in the respective systems and placed in Petri dishes for 18 h. The cleaning efficiency, estimated as the weight percent of greasy soil removal, is reported in Figure 12.

**Figure 12:**
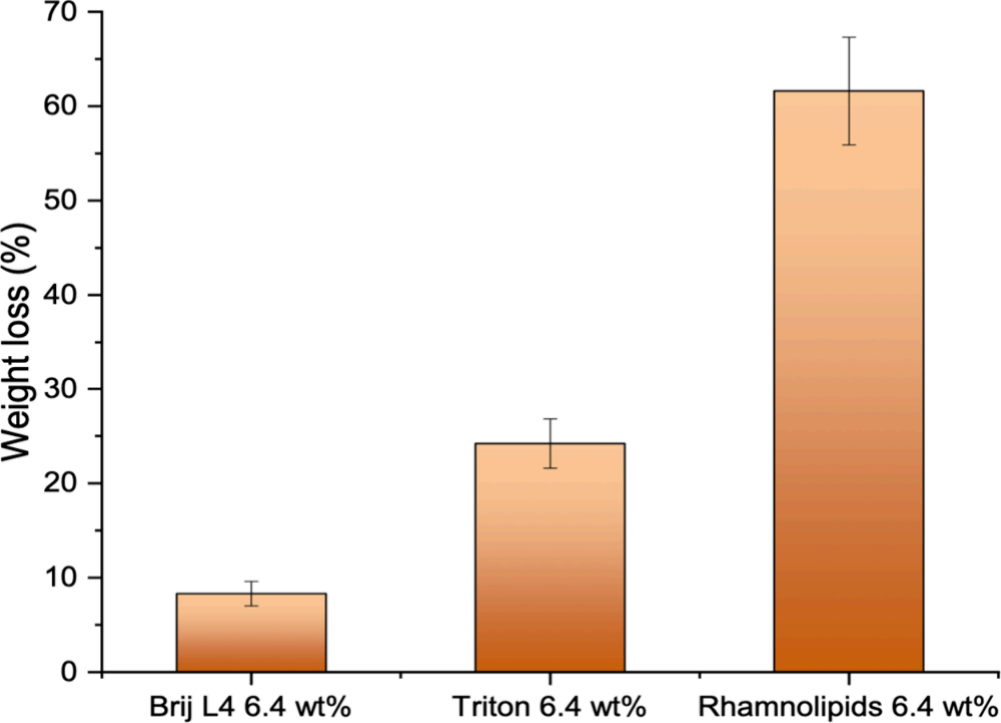
Cleaning efficiency comparison of three different class surfactants at the same active concentration.

The water–Rha mixture demonstrated the highest cleaning efficiency, approximately 63 wt %, surpassing commercial nonionic ethoxylated (Brij L4) and glycosylated (Triton CG-110) surfactants. At this surfactant concentration, Triton CG-110 forms small spherical micelles,^59^ which are unable to effectively solubilize oils. On the other hand, Brij L4 forms rod-like aggregates^60^ whose presence increases viscosity, which is detrimental to soil removal. Thus, it seems that the medium-size ellipsoidal micelles formed by Rha, whose dimensions are not affected by the concentration and result in low viscosity, are the best compromise for effective soil removal. A synergistic effect could be played by the external layer of the Rha aggregates, stabilized by a large number of H-bonds that are formed between rhamnose units and carboxylic groups.^10^ These H-bonds may also involve the free fatty acids, thereby contributing to the compactness of the aggregate, thus enhancing the capacity to solubilize contaminants and reducing soil redeposition.

## 4 Conclusions

Rhamnolipids exhibit a rich chemodiversity combined with complex molecular architectures. Congeners present in commercial samples may differ in a variety of structural features, resulting in a large number of possible combinations. These samples also may contain other metabolites derived from bacterial fermentation. The self-aggregation behavior of these mixtures in water is contingent on the specific combination of different congeners and the nature of their intermolecular interactions.

In this context, this work consists of two main parts. In the first part, NMR, GC-MS, and HPLC-MS measurements were combined to determine the qualitative and quantitative composition of a commercial sample of rhamnolipids, termed Rha for simplicity. Specifically, an NMR-based method was developed to determine the percentages of mono- and dirhamnolipids, as well as of single- and double-tailed species, present in Rha. It was also able to reveal the presence of free fatty acids. This method did not require sample pretreatment or purified standards, establishing itself as a relatively direct and reliable method for investigating the mixture composition. Overall, most congeners present two rhamnose units and two acyl tails. The results of the GC-MS and HPLC-MS analyses enabled a more thorough examination of the individual congeners present, showing that the C10 tail is largely prevalent. Importantly, GC-MS revealed the presence of long-chain free fatty acids (up to approximately 20 wt %) and indicated the presence of unsaturated and branched tails, albeit in small amounts.

The second part of this study combined visual inspection, POM, SAXS, EPR, and rheology experiments to investigate the aggregation and flow behavior of Rha in water. These techniques all converged in showing the formation of an isotropic solution up to a very high Rha content. With a further concentration increase, a transition to anisotropic structures was observed. However, the threshold between these two conditions varied depending on the technique. Water–Rha samples flow freely under their own weight up to 75 wt % Rha, with gel formation observed macroscopically only at 80 wt %. This threshold limit seems to be confirmed by EPR spectra, which show a sudden transition and a dramatic change in molecular organization, as well as by the onset of viscoelastic behavior, as detected by rheology. Conversely, POM images reveal that the samples exhibit birefringence, indicative of optically anisotropic structure formation, beginning at 70 wt %, which is also the concentration at which viscosity measurements deviate from Newtonian behavior. Finally, the SAXS profiles exhibit narrow peaks, characteristic of ordered structures, beginning at 75 wt %. It is important to note that neither POM nor SAXS succeeded in identifying a single structure, whether in the form of LLCs or solid crystals in concentrated solutions. This suggests the possible coexistence of different structures or their inability to expand to a significantly large length scale. The presence of congeners with different molecular features and free fatty acids, acting as “defects”, hinders the growth of structured domains. The increasing number of ordered local domains is progressively detected by the other techniques depending on their sensitivity. Thus, the presence of different congeners plays a key role in the structure and dynamics of concentrated Rha mixtures, resulting in complex behavior.

Since the main purpose of this work was to verify the possibility of using rhamnolipids for the formulation of ultraconcentrated liquid products, it is important to conclude by focusing on the characterization of the isotropic liquid solution that forms up to at least 65 wt %. In this range, prolate ellipsoidal micelles form, whose morphological and structural features barely change with increasing concentration, resulting in low-viscosity systems. This aggregation behavior is led by the predominant species, which are double-tailed dirhamnolipids. However, an important role is also played by the other components present in the Rha mixture. Specifically, free fatty acids act as cosurfactants, leading to bulkier aggregates with a more hydrophobic inner core. Moreover, branched fatty acids may also contribute to increasing the hydrophobicity of the aggregate’s inner core. Thus, the composition of the Rha mixture, both predominant and less represented components, concur in determining the aggregation behavior. The formation of medium-sized ellipsoidal micelles with a highly hydrophobic core yet maintaining low viscosity could be the key to interpreting the very encouraging cleaning test results. This makes rhamnolipids ideal to be used in concentrated, surfactant-based products, establishing them as pivotal ingredients in ecofriendly, innovative formulations.

## Supplementary Material



## Notes

Experimental procedures, pseudo-2D DOSY spectrum (Figure S1), NMR assignment of the Rha (Figure S2), GC-MS data (Figures S3 and S4), visual and POM analyses of selected samples (Figure S5), SAXS profiles reported on a linear *q* scale (Figure S6), positions of peaks in the SAXS profiles (Table S1), and SAXS parameters at different temperature (Table S2) supporting the results discussed in the main text (PDF)
