# Supplementary material for: Concentrated Rhamnolipid Formulations: Bridging Chemodiversity to Structure, Flow Behavior, and Functionality
Source: ACS Sustain Chem Eng. 2025 Dec 17;14(1):415–27. doi: 10.1021/acssuschemeng.5c09437 (PMC12801974; doi:10.1021/acssuschemeng.5c09437)
Supplement: Supplementary file 1 [file sc5c09437_si_001.pdf]

Supporting Information for the Article

Concentrated Rhamnolipid Formulations: Bridging Chemodiversity to Structure, Flow Behavior and Functionality

Matilde Tancredi[a,b], Carlo Carandente Coscia[a,b], Michela Buonocore[a], Alessandro Cangiano[a,b], Maria Michela Salvatore[c], Lorenzo Veronico[d], Delia Picone[a], Anna Maria D'Ursi [e], Manuela Grimaldi [e], Maria Francesca Ottaviani[f], Stefano Guido[g], Luigi Paduano[a,b], Luigi Gentile[b,d], Gerardino D'Errico[a,b,*]

[a] *Department of Chemical Sciences, University of Naples Federico II, Complesso Universitario di Monte Santangelo, Via Cintia 4, I-80126 Naples, Italy*

[b] *Consorzio Interuniversitario per lo Sviluppo dei Sistemi a Grande Interfase (CSGI), Via della Lastruccia 3, I-50019 Florence, Italy*

[c] *Department of Veterinary Medicine and Animal Production, University of Naples Federico II, Via Federico Delpino 1, I-80137 Naples, Italy*

[d] *Department of Chemistry, University of Bari "Aldo Moro", Via Orabona 4, I-70126 Bari, Italy*

[e] *Department of Pharmaceutical Sciences, University of Salerno, via Ponte Don Melillo, I-84084 Fisciano, Italy*

[f] *Department of Pure and Applied Sciences, University of Urbino "Carlo Bo", Via Saffi 2, I-61029 Urbino, Italy*

[g] *Department of Chemical, Materials and Production Engineering, University of Naples Federico II, P.le Tecchio 80, I-80125 Naples, Italy*

Number of pages: 13

Number of figures: 6

Number of tables: 2

* Corresponding author.
E-mail address: gerardino.derrico@unina.it (G. D'Errico)

S1 Materials and Methods

S1.1 Materials

A commercial rhamnolipid mixture from the strain *P. aeruginosa* (Rha, 85–90% pure in solid/granular form and brownish in color) was purchased from AGAE Technologies (AGAE Technologies, LLC, Corvallis, OR, USA) . Deuterium oxide (D_2O, deuteration degree ≥99.90% for NMR spectroscopy), trimethylsilylpropanoic acid (TSP, deuteration degree ≥98.0%), ethyl acetate (EtOAc, purity ≥99.9% for HPLC analysis), acetonitrile (purity ≥99.9% for HPLC analysis), hydrochloric acid (HCl, diluted solution with concentration 37.0%), sodium sulfate anhydrous (Na_2SO_4, purity ≥99.0%), palmitic acid (purity ≥99.0% analytical grade standard), *N,O*-bis(trimethylsilyl)trifluoroacetamide (BSTFA, purity ≥99.0% for GC analysis), 2-(14-carboxytetradecyl)-2-ethyl-4,4-dimethyl-3-oxazolidinyloxy (16-DOXYL-stearic acid free radical, 16-DSA), ethanol (EtOH, purity ~96%) were purchased from Merck (KGaA, Darmstadt, Germania). Helium (He, purity ≥ 99.9999%) was supplied by Sol spa (Monza, Italy). Unless otherwise stated, ultrapure deionized water from a Millipore Milli-Q system with an electrical conductivity of less than 1×10^{-6} S cm^{-1} at 25 °C was used as the solvent.

S1.2 Analysis of the Rha composition

S1.2.1 NMR spectroscopy. NMR experiments were performed on samples prepared as 10% w/w solutions in D_2O. TSP was added to a final concentration of 1 mM as an internal standard. All spectra were acquired on a Bruker Avance 600 MHz (Rheinstetten, Germany) spectrometer equipped with a 5 mm triple resonance $^{1}H(^{13}C/^{15}N)$, z-axis pulsed-field gradient probe head. For the characterization and assignment of rhamnolipids, 1D ^{1}H spectra were acquired using the zg pulse sequence. Two-dimensional ^{1}H-^{1}H correlation spectroscopy (COSY) experiments (cosyphpr) were recorded with a spectral width of 12 ppm in both dimensions, a relaxation delay of 2 s and 32 scans per increment. ^{1}H-^{13}C heteronuclear single quantum coherence (HSQC) spectra were acquired using the hsqcedetgpsisp2 sequence with spectral widths of 12 ppm for ^{1}H and 180 ppm for ^{13}C, a relaxation delay of 2 s, and 32 scans per increment. HSQC-total correlation spectroscopy (TOCSY) experiments (hsqcdiedetgpsisp.2) were recorded with a mixing time of 80 ms using the same spectral widths and acquisition parameters as HSQC. For long-range correlations, ^{1}H-^{13}C heteronuclear multiple bond correlation (HMBC) spectra (hmbcgplpndqf) were acquired with spectral widths of 12 ppm for ^{1}H and 220 ppm for ^{13}C, a relaxation delay of 2 s and 32 scans per increment.

Pseudo-2D diffusion-ordered spectroscopy (DOSY) experiments (stebpgp1s) were performed to assess the diffusion behavior of the rhamnolipid species and other species, using a gradient pulse length (δ) of 2.5 ms, a diffusion delay (Δ) of 150 ms, 32 gradient steps with a linear ramp from 2% to 95% of the maximum gradient strength, 16 scans per gradient, and a spectral width of 12 ppm. The spectra were processed and analyzed using the Dynamic Center software included in TopSpin 4.4.0 (Bruker).

For the quantitative analysis of the carbon signals, 1D ^{13}C NMR spectra were acquired using the zgig30 sequence, which suppresses the nuclear Overhauser effect (NOE) by turning off the decoupling field during the relaxation delay. The parameters used included a relaxation delay (d1) of 10 s, a spectral width of 220 ppm, 64k data points, and 512 scans to ensure reliable quantitative integration. All spectra were processed using Bruker TopSpin 4.4.0 and MestreNova 9 (MestreLab Research S.L., Santiago de Compostela, Spain).

S1.2.2 LC-MS analysis. A weighted amount of Rha was dissolved in water (5 mL, Rha content ~1.5 wt%), acidified to pH ~ 2, and extracted three times with the same volume of EtOAc. Organic phases were combined, dried on Na_2SO_4 and evaporated under reduced pressure. The extract was dissolved in H_2O:acetonitrile (2:3, v/v) and analyzed by an Agilent LC-MS electrospray ionization time-of-flight (ESI-TOF) 1260/6230DA (Cernusco sul Naviglio, Milan, Italy) instrument operating in negative ionization mode. The source temperature was kept at 120 °C and the desolvating gas at 250 °C. The instrument was interfaced to an Agilent Eclipse Plus ODS column (150×4.6 mm, 5 µm). An acetonitrile-water gradient was used. The elution was started with 40%

acetonitrile for 4 min and the acetonitrile concentration was raised to 90% after 20 min. HPLC flow rate was 400 μL/min.

S1.2.3 GC-MS analysis. In Experiment 1, an aliquot of Rha was hydrolyzed. Briefly, a weighted amount of Rha was dissolved and refluxed in 2 M HCl (5 mL, Rha content ~1.5 wt%) for 2 h. After this treatment, the solution was extracted with the same volume of EtOAc three times. The organic extracts were combined, dried on Na_2SO_4, and evaporated under reduced pressure. The residue was trimethylsilylated with BSTFA and presented to the GC-MS. In Experiment 2, a weighted amount of Rha was dissolved in Millipore deionized water (5 mL, Rha content ~1.5 wt%), acidified to pH ~ 2, and extracted three times with the same volume of EtOAc. Organic phases were combined, dried on Na_2SO_4 and evaporated under reduced pressure.

The residue was derivatized with BSTFA and analyzed by GC-MS. Experiments were performed in triplicate. Trimethylsilyl derivatives were analyzed by an Agilent 6850 GC (Cernusco sul Naviglio, Milan, Italy), equipped with an HP-5MS capillary column (5% phenyl methyl polysiloxane stationary phase), coupled to an Agilent 5973 Inert MS detector operated in the full scan mode (m/z 35–550) at a frequency of 3.9 Hz and with the Electron Ionization (EI) ion source and quadrupole mass filter temperatures kept, respectively, at 200 and 250 °C. Helium was used as carrier gas at a flow rate of 1 mL·min^{-1}. The injector temperature was 250 °C and the temperature ramp raised the column temperature from 70 to 280 °C: 70 °C for 1 min; 10 °C·min^{-1} until reaching 170 °C; and 30 °C·min^{-1} until reaching 280 °C. Then, it was held at 280 °C for 5 min. The solvent delay was 4 min.

The identification of fatty acids was performed by matching their EI mass spectra at 70 eV with those stored in the NIST 20 mass spectral library (https://www.nist.gov/srd/nist-standard-reference-database-1a). Furthermore, the identification was supported by the Kovats retention index (RI) calculated for each metabolite by the Kovats equation using the standard n-alkane mixture in the range C7–C40 (Sigma-Aldrich, Saint Louis, MO, USA) analyzed under the same conditions. To compensate for the effects on peak areas of extensive variables and random factors that may influence measurements made in different experiments at different times, palmitic acid (chromatographic peak at about 13.5 min) was selected as a housekeeping internal standard so that, by dividing fatty acids peak areas by the palmitic acid peak area, a normalized area was finally assigned to each detected fatty acid in each chromatogram.

S1.3 Analysis of the aggregation and flow behavior of water-Rha mixtures

S1.3.1 Sample preparation. Samples for POM, SAXS, rheology, and EPR measurements were prepared by weighing appropriate amounts of Rha and Millipore deionized water o obtain mixtures with weight-to-weight percentages ranging from 20% to 90%. Different sets of samples were prepared, with at least three independent samples prepared for each concentration. The samples were placed in either screw-cap or round-bottom vials (12 mm and 8 mm o.d., respectively). The latter were flame-sealed immediately. The mixtures were vortexed and then subjected to low-speed centrifugation at approximately 400 rpm. Several centrifugation steps were performed by alternately inverting the sample orientation. All the samples were subjected to thermal cycles between low and high temperatures (~4 °C and ~80 °C, respectively). Prior to analysis, all samples were equilibrated and stored at 25 °C for a minimum of one week. No visual variation was observed subsequent to the equilibration period, nor was there a difference between samples prepared in sealed ampoules and screw-cap vials. Rheological measurements were acquired for selected samples both after one week and after three months. Negligible variations were found, thus confirming that the one-week equilibration time is sufficient. The pH of the diluted samples (less than 10 wt% Rha) was measured to be around 6, which is close to the pKa of the carboxylic group of the self-aggregated rhamnolipids. Since high concentrations depress weak acid dissociation, the rhamnolipids were considered to be predominantly in their undissociated form. For EPR analysis, an aliquot of 3 μL of EtOH-dissolved spin probe 16-DSA, 1 mg mL^{-1}, was added to 0.5 g of each equilibrated mixture. The inclusion of the spin probe and the presence of a small amount of ethanol in the samples were checked to ensure they would not affect the EPR spectra. This was verified for selected samples by changing the probe concentration and/or evaporating the ethanol from the

probe solution at the bottom of a round vial before adding the Rha aqueous mixture. The spin-probed sample was subjected to a second round of mixing and allowed to rest for a minimum of two days. Subsequently, 50 µL of each equilibrated spin-probed sample was transferred into glass capillaries and flame-sealed.

S1.3.2 Polarized optical microscopy. Images were collected using a Axiovert 200 M or a Cell Observer light microscope (Carl Zeiss Light Microscopy, Germany) and a home-made incubator capable of maintaining a constant sample temperature within 0.1 °C. A small amount of water-Rha mixtures at different concentrations was squeezed between a glass microscope slide and a coverslip. To control the sample thickness, a double-sided adhesive tape was placed between the two glass surfaces as a spacer, resulting in a thickness of 130 µm. Observations were made between crossed polarizers. Representative images were recorded using an AxioCam HRm high-resolution digital camera.

S1.3.3 Small-angle X-ray scattering. SAXS experiments were conducted at the Diamond Light Source B21 Beamline (Didcot, United Kingdom). The beamline configuration consisted of a beam energy of 13.018 keV and a sample-to-detector distance of 3.7 m. Using this setup, data were collected for the scattering vector modulus q=4 π sin(θ/2) λ between 0.0045 Å^{-1} and 0.34 Å^{-1}, where θ is the scattering angle, with a resolution estimated to be about 0.003 Å^{-1}. Samples were placed in quartz capillaries with an outside diameter of 2 mm and a wall thickness of 0.01 mm (WJM-Glas, Berlin, Germany). SAXS data were collected in the temperature range 20 °C – 50 °C with 10 °C temperature increase steps.

Experimental data of the samples containing up to 50 wt% Rha were fitted using the SASView v5.0.6 Software (www.sasview.org) using a custom module made of the combination of the Power Law and the Core-Shell Ellipsoid models.

$$I(q) = \text{scale} \cdot q^{-\text{power}} + \frac{\text{scale}}{V} F^2(q) + \text{background} \tag{1}$$

For the Core-Shell Ellipsoid model $F(q)$ is:

$$F(q) = \frac{3\,\Delta\rho\, V\left(sin\left[q\left(R_\text{e}^2+R_\text{p}^2\right)^{1/2}\right] - cos\left[q\left(R_\text{e}^2+R_\text{p}^2\right)^{1/2}\right]\right)}{\left(q\left(R_\text{e}^2+R_\text{p}^2\right)^{1/2}\right)^3} + f(q, R_\text{e} + t_\text{shell}, R_\text{e} r_\text{core} + t_\text{shell}, r_\text{shell}) \tag{2}$$

where $V = \frac{4}{3}\pi\, R_\text{p}\, R_\text{e}^2$ is the volume of the ellipsoid, and R_p and R_e are the polar and the equatorial radius respectively. $\Delta\rho$ is the contrast, t_shell is the equatorial thickness of the shell, and r_core and r_shell are the core polar radius and the polar thickness of the shell, respectively.

S1.3.4 Electron paramagnetic resonance. EPR spectra were acquired at room temperature (25 ± 2 °C) using a Bruker Elexsys E-500 X-band spectrometer (9.87 GHz, Rheinstetten, Germany). The flame-sealed glass capillaries containing the spin-probed samples were placed in 4mm quartz EPR tubes. These tubes were then inserted into the super-high-sensitivity probe head of the spectrometer. The following parameters were fixed for the spectra acquisition: microwave power 6.40 mW, sweep width 140 G, center field 3500 G, 1024 data points, time constant 10.24 ms, conversion time 20.48 ms, modulation frequency 100 kHz, and modulation amplitude 1.0 G. To improve signal-to-noise ratio, up to 16 scans were accumulated per spectrum.

The EPR spectra of 16-DSA spin probes in water–rhamnolipid mixtures were simulated. To simplify the calculations and based on the known magnetic properties of the doxyl group, the components of the g tensor were fixed at g_{xx} = 2.009, g_{yy} = 2.006, and g_{zz} = 2.003. Similarly, the A tensor values related to the hyperfine interaction between the unpaired electron and the nitrogen nucleus were kept constant at A_{xx} = A_{yy} = 6 G, while A_{zz} was adjusted to best fit the experimental spectra. For the simulation of each spectrum, other parameters were optimized depending on the specific spectrum features.

S1.3.5 Rheology. The rheological properties of the system were characterized using an MCR302 evolution stress-controlled rheometer (Anton Paar GmbH, Graz, Austria), equipped with a Taylor-Couette (concentric cylinder) geometry. The geometry had an inner diameter of 16.662 mm and a sample gap of 0.704 mm. Temperature control was achieved via a Peltier system, accurate to ±1 °C, with a water-bath circulator serving as a reference.

Viscosity (η) measurements as a function of shear rate ($\dot{\gamma}$) (flow curves) were conducted on the water-Rha mixtures over a concentration range from 10 wt% to 90 wt% at temperatures of 15 °C, 25 °C, 37 °C, and 45 °C. The shear rate varied from 0.1 to 100 s^{-1}. Additionally, small amplitude oscillatory shear (SAOS) tests were performed at a strain of 0.1% which had been preventively checked to be in the linear viscoelastic regime. These tests provided the storage modulus (*G′*) and the loss modulus (*G″*) as functions of the oscillatory angular frequency (ω). All rheological experiments were performed on three independent replicates (*n* = 3).

S1.4 Cleaning efficiency test

Cleaning efficiency was assessed using a simplified procedure based on recommendations from IKW (Industrieverband Körperpflege und Waschmittel), the German Cosmetics, Toiletries, Perfumes and Detergents Association, for cleaning performance.

This method measures the amount of oil that the surfactant mixture can absorb from a surface. A stainless-steel tile was pretreated with a solution of potassium hydroxide (0.08% by weight/volume) and ethanol. At this point, a small amount of a model greasy soil was spread on its surface. The greasy soil was a 4:1 mixture of oils and HSW soil by weight. The oil mixture consisted of peanut, sunflower, and corn oils, while the HSW soil consisted primarily of humus, cement, silica gel, and clay. This mixture was designed to mimic used cooking oil. The soiled tile was artificially aged by placing it in an oven at 135 °C for two hours. At this temperature most edible and technical oils begin to oxidize in the presence of oxygen, resulting in the formation of oil-soluble acids, resins, and varnish-like byproducts. After cooling, the tile was utilized for the test. The soiled tile was weighed using an analytical balance. Then, it was placed in a Petri dish and covered with the surfactant mixture to be tested for 18 h. Afterwards, it was weighed again. The cleaning efficiency was evaluated as

$$weight\ loss\ \% = 100\,\frac{w_i - w_f}{w_i - w_t} \tag{4}$$

where w_t represents the weight of the clean tile, w_i and w_f are the weights of the soiled tile before and after the test, respectively. Three independent experiments were performed for each considered surfactant.

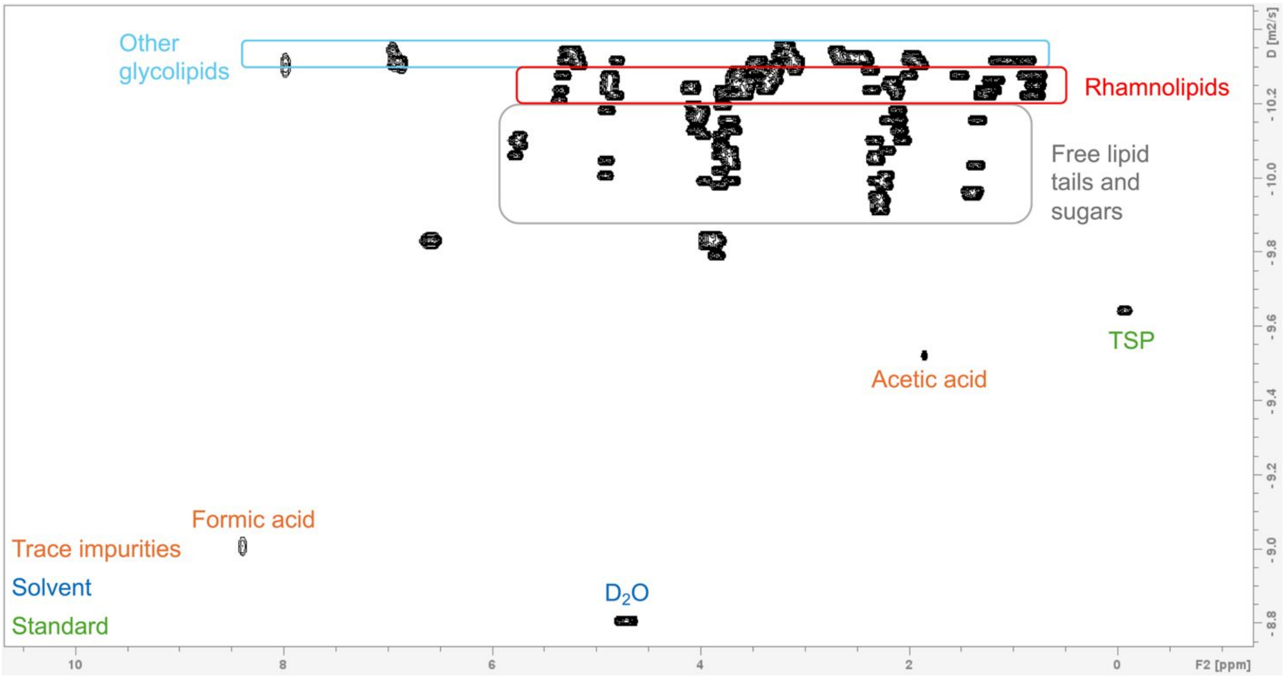

Figure S1. Pseudo 2D DOSY spectrum correlating the ^1H chemical shifts (F2, ppm) with the diffusion values (D, m^2/s, Log_{10} scale) acquired on the sample in D_2O. The rhamnolipids self-diffusion value corresponds to a micelle hydrodynamic radius of 3.5 nm, consistent with the radius of the volume-equivalent sphere calculated from the ellipsoid semi-axes obtained by SAXS (3.3 nm).

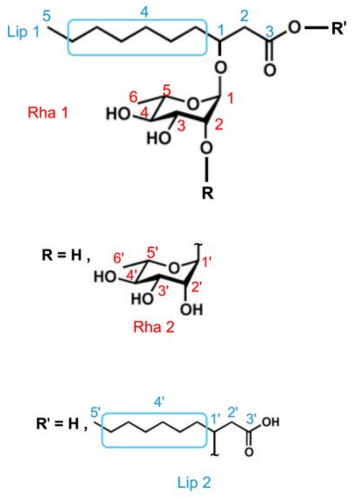

Mono-rhamnolipids

Group	Atom	^{1}H (ppm)	^{13}C (ppm)
Rha 1	1	**4.89**<br>**4.88**	**97.28**<br>**98.19**
	2	**3.88**<br>**3.85**	**70.89**<br>**70.91**
	3	3.64-3.91	67.1-76.7
	4	3.64-3.91	67.1-76.7
	5	3.64-3.91	67.8-74.1
	6	1.27	16.86

Di-rhamnolipids

Group	Atom	^{1}H (ppm)	^{13}C (ppm)
Rha 1	1	**4.96**<br>**4.93**	**96.08**<br>**96.67**
	2	**3.86**<br>**3.82**	**78.07**<br>**78.59**
	3	3.64-3.91	67.1-76.7
	4	3.64-3.91	67.1-76.7
	5	3.64-3.91	67.8-74.1
	6	1.27	16.86
Rha 2	1'	**4.95**<br>**4.94**	**102.44**<br>**102.52**
	2'	**4.07**	**70.13**
	3'	3.64-3.91	67.1-76.7
	4'	3.64-3.91	67.1-76.7
	5'	3.64-3.91	67.8-74.1
	6'	1.27	16.86

Single-tailed rhamnolipids

Group	Atom	^{1}H (ppm)	^{13}C (ppm)
Lip 1	1	**4.11**	**75.07**
	2	**2.38**	**43.56**
	3	-	**180.47**
	4	1.10 – 1.61	12.85 – 46.21
	5	0.86	14.03

Bi-tailed rhamnolipids

Group	Atom	^{1}H (ppm)	^{13}C (ppm)
Lip 1	1	**4.12**	**73.31**
	2	**2.61**	**40.04**
	3	-	**172.23**
	4	1.10 – 1.61	12.85 – 46.21
	5	0.86	14.03
Lip 2	1'	**5.28**	**73.46**
	2'	**2.50**	**42.73**
	3'	-	**178.13**
	4'	1.10 – 1.61	12.85 – 46.21
	5'	0.86	14.03

Figure S2. NMR assignment of the rhamnolipid species found in the extract under examination. The characteristic signals for each species are reported in bold.

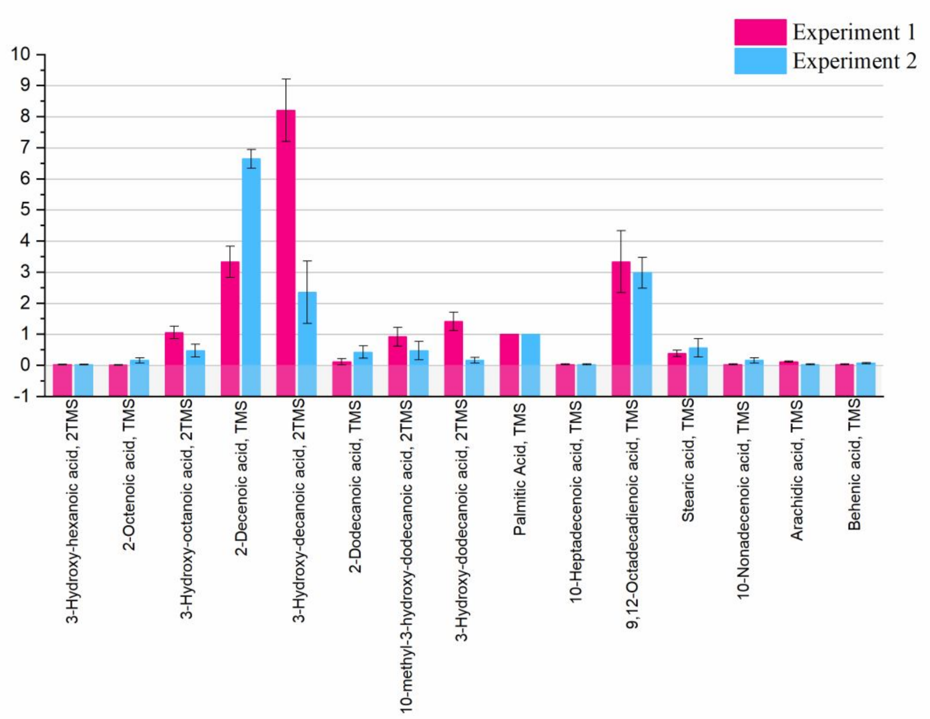

Figure S3. Normalized areas of fatty acids peaks measured from GC-MS chromatograms acquired in Experiment 1 (orange bars) and Experiment 2 (blue bars) experiments. Reported values are averages of three independent experiments and error bars represent 95% confidence intervals. TMS represents the trimethylsilyl function ($(CH_3)_3Si$–) and red labels indicate 3-hydroxy fatty acids.

According to the logic which connects Experiment 1 to Experiment 2, saturated and unsaturated fatty acids (which lack the structural features to participate in rhamnolipids formation) should expose, within experimental uncertainty, the same normalized areas both in Experiment 1 and Experiment 2 chromatograms. Figure S3 shows that this prediction is generally satisfied, but 2-decenoic acid and 2-dodecenoic acid seem to overturn the chemical logic behind our experiments because they expose in Experiment 2 chromatograms normalized areas significantly larger than in Experiment 1 chromatograms.

To explain this unexpected occurrence, it should be considered that direct extraction of the whole non-hydrolyzed mixture with EtOAc performed in Experiment 2, does not extract exclusively free fatty acids (as desired) but also rhamnolipids which are soluble in ethyl acetate. Because of this, BSTFA derivatization of Experiment 2 extracts will convert both free acids and rhamnolipids to their trimethylsilyl derivatives. As expected, when this mixture of derivatized fatty acids and rhamnolipids is injected in the GC, the high temperature of the injector (250 °C) will decompose the derivatized rhamnolipids which cannot be detected by GC-MS. Thus, it could be suggested that the excess of 2-decenoic acid and 2-dodecenoic acid detected in EtOAc extracts from the non-hydrolyzed mixture are produced by a specific decomposition reaction of silylated rhamnolipids which is promoted by the high temperature of the GC injector. For instance, the hydrogen transfer reaction represented in Figure S2 is a possible mechanism of decomposition which may explain the above anomalies in Experiment 2 chromatograms. Finally, whatever may be the mechanism of decomposition of rhamnolipids in the GC injector, since a trimethylsilylated 2-unsuturated fatty acid can only be produced from the terminal hydroxy acid of silylated rhamnolipids (Figure S4), the increase in the response of 2-octenoic, 2-decenoic and 2-dodecenoic acids in Experiment 2 is the unambiguous

manifestation of the fact that both rhamnolipids with terminal 3-hydroxy-octanoic, 3-hydroxy-decanoic and 3-hydroxy-dodecanoic acid are present in the investigated mixture.

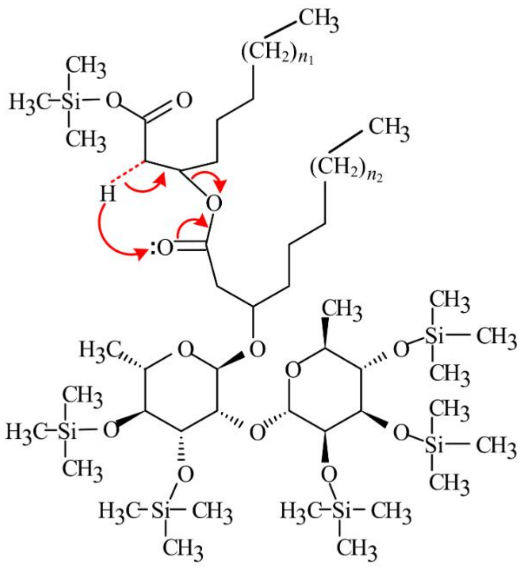

Figure S4. Conceptual representation of the decomposition of a dirhamnolipid through hydrogen transfer and liberation of the terminal 3-hydroxy acid in the form of the corresponding trimethylsilylated 2-unsuturated fatty acid.

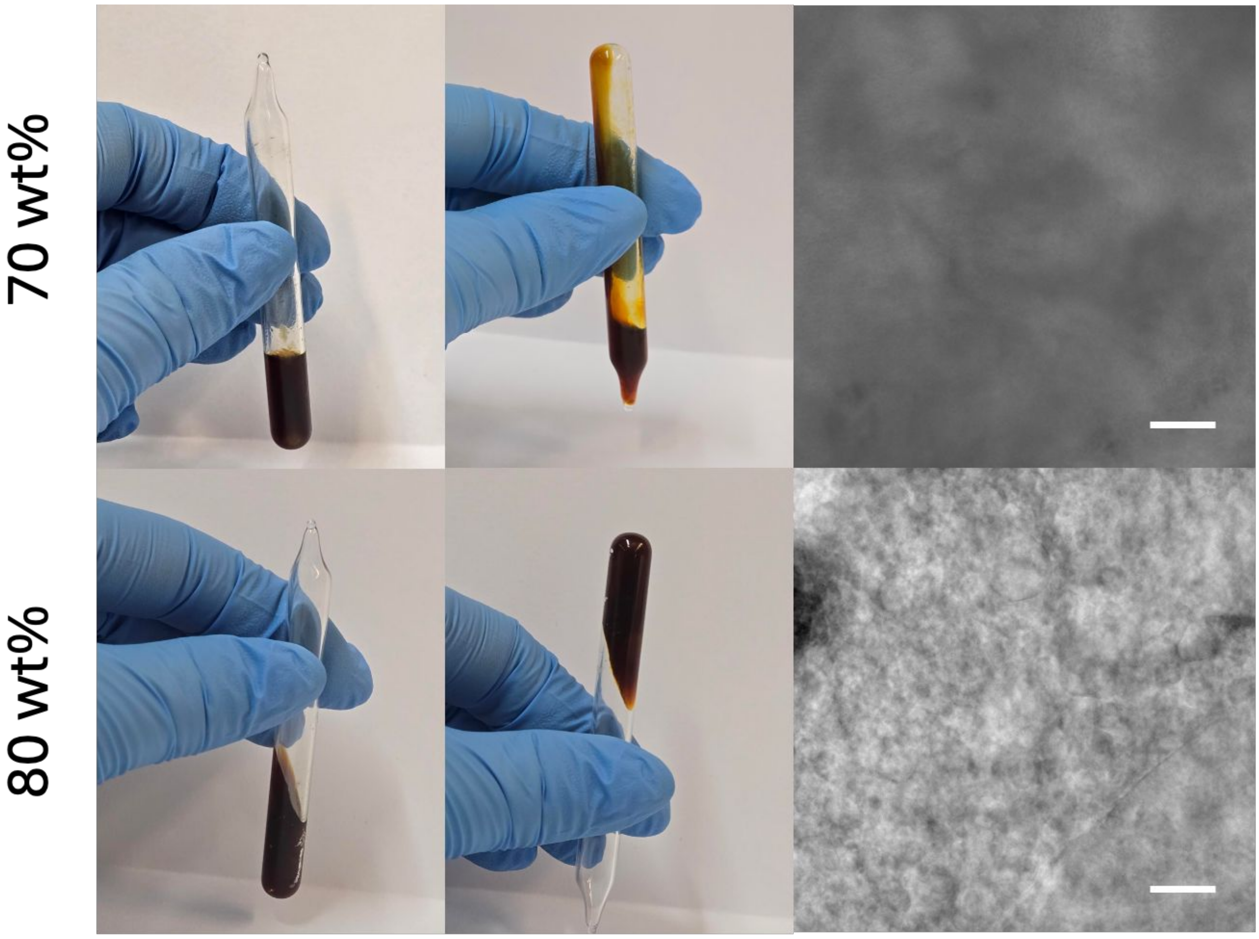

Figure S5. Visual and POM analysis of selected water-Rha mixtures at 25 °C. The top images are photographs of the samples taken 5 s after tilting. The bottom images display representative POM images under crossed polarizers. The scale bar is 100 μm. The oblique free surface of the 80 wt% sample is due to the orientation of the test tube inside the centrifuge used for mixing.

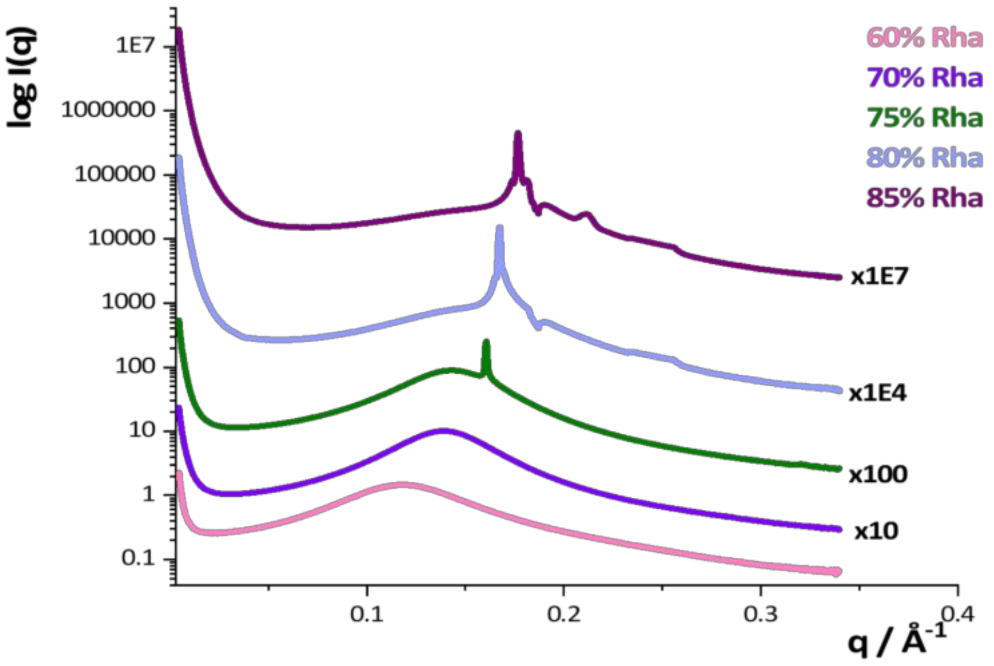

Figure S6. SAXS profiles of water-Rha mixtures at concentrations ranging from 60 to 85 wt% at 20 °C.

Table S1. Positions of the additional peaks observed in the SAXS profiles for the water-Rha mixtures at 80 and 85 wt% at 20 °C.

Sample	q / $Å^{-1}$
80 wt% Rha	0.1695<br>0.1732<br>0.1820
85 wt% Rha	0.1776<br>0.1815<br>0.1844<br>0.2115

Table S2. Ellipsoidal aggregates structural parameters obtained from the fitting of the SAXS data for the water-Rha mixtures from 30 °C to 50 °C.

Concentration	T (°C)	r_{core}/r_e	t_{shell}/r_{shell}	t_{shell} (Å)	r_e (Å)
10 wt% Rha	30	3.9	1.1	15.1 ± 1.0	10.8 ± 0.5
	40	4.0	1.1	14.5 ± 0.8	11.2 ± 0.2
	50	4.2	1.1	13.8 ± 1.0	11.0 ± 1.0
20 wt% Rha	30	4.1	1.1	14.8 ± 1.0	11.3 ± 0.5
	40	3.8	1.1	15.0 ± 0.5	11.0 ± 1.0
	50	4.0	1.1	15.2 ± 0.5	10.8 ± 0.5
30 wt% Rha	30	4.1	1.1	13.9 ± 0.5	11.2 ± 1.0
	40	3.9	1.1	14.5 ± 1.0	12.0 ± 1.0
	50	4.0	1.1	15.5± 1.0	10.2 ± 1.0
40 wt% Rha	30	4.0	1.1	15.6 ± 1.0	10.3 ± 0.7
	40	3.8	1.1	15.2 ± 0.4	11.0 ± 0.3
	50	4.0	1.1	15.3 ± 0.5	10.0 ± 1.0
50 wt% Rha	30	4.0	1.1	15.3 ± 0.5	10.0 ± 1.0
	40	4.2	1.1	16.0 ± 1.0	10.5 ± 0.5
	50	4.0	1.1	15.5 ± 1.0	11.0 ± 0.5
